# Total flavonoids of *Rhizoma drynariae* targets NRF2-mediated anti-ferroptosis in osteoblasts to promote induced membrane osteogenesis

**DOI:** 10.1186/s13020-026-01347-7

**Published:** 2026-03-13

**Authors:** Shuyuan Li, Dawen Yang, Zhanpeng Zeng, Qunbin Cai, Qishi Zhou

**Affiliations:** The First Affiliated Hospital of Guangzhou University of Chinese Medicine, Baiyun district airport road number 16, Guangzhou, Guangdong China

**Keywords:** Total flavonoids of *Rhizoma drynariae*, Induced membrane technique, NRF2, Ferroptosis, Bone defect

## Abstract

**Background:**

Induced membrane technique (IMT), a novel approach for reconstructing critical-size bone defect, encounters the challenge of lengthy mineralization time after bone grafting. Total flavonoids of *Rhizoma drynariae* (TFRD), the extracts from dried rhizome of Drynaria roosii Nakaike, is widely used in the treatment of orthopedic diseases.

**Purpose:**

This study primarily investigates the impact of TFRD on the NRF2-mediated anti-ferroptosis effect in osteoblasts within the IMT bone grafting area.

**Methods:**

An IMT model was established in the right femur of rats. After 4 and 8 weeks of treatment with TFRD and DMF (an NRF2 activator) respectively, bone defect repair and ferroptosis-related indicators were evaluated. In vitro, an Erastin-induced ferroptosis model of osteoblasts was constructed to analyze the mineralization capacity of osteoblasts, ferroptosis-related indicators, and factors related to the NRF2/ARE pathway under TFRD and DMF treatment. Additionally, the components of TFRD and TFRD-containing serum were analyzed using UHPLC-Q-Orbitrap HRMS. Finally, the main compounds in TFRD that bind to the NRF2 protein were studied through molecular docking, molecular dynamics simulation (MDS), and CETSA.

**Results:**

In vivo results demonstrated that excessive iron accumulation occurred in the IMT bone grafting area, accompanied by elevated levels of lipid peroxidation products (MDA and 4-HNE) and decreased levels of antioxidants (GSH), suggesting the presence of ferroptosis during the bone graft mineralization process in IMT. Treatment with TFRD and DMF reduced iron accumulation and the production of MDA and 4-HNE, accelerated bone defect healing, and enhanced expression of osteogenesis-related factors and NRF2/ARE pathway factors. In vitro experiments revealed that Erastin induced ferroptosis in osteoblasts, diminishing cell viability and mineralization capacity. Treatment with TFRD and DMF alleviated mitochondrial damage, reduced production of ROS, MDA and 4-HNE, increased the expression of osteogenesis-related factors, upregulated the NRF2/ARE pathway, and enhanced cell viability and mineralization. Meanwhile, five active components in TFRD and TFRD-containing serum were identified using UHPLC-Q-Orbitrap HRMS. Molecular docking, MDS and CETSA results indicated that the main compounds in TFRD-containing serum could directly bind to the NRF2 protein in osteoblasts and maintain its stability.

**Conclusion:**

The NRF2-mediated anti-ferroptosis effect in osteoblasts positively regulated the mineralization of osteoblasts. The main components of TFRD targeted NRF2 in osteoblasts within the IMT bone grafting area, maintained its stability, promoted the expression of downstream antioxidant response elements (ARE), enhanced the anti-ferroptosis role of osteoblasts, thereby accelerating the repair of bone defects.

**Supplementary Information:**

The online version contains supplementary material available at 10.1186/s13020-026-01347-7.

## Introduction

The repair of large-segment bone defects presents a significant clinical challenge. Traditional autologous bone grafting is considered the "gold standard" for treating bone defects, but it is associated with issues such as donor-site complications and limited bone graft quantity [1]. Allogeneic bone grafting, on the other hand, faces risks of immune rejection and disease transmission [2]. In recent years, bone repair strategies based on the induced membrane technique (IMT, also known as Masquelet technique) have garnered considerable attention due to their simple operation and relatively high osteogenic efficiency. This technique involves a two-stage surgical procedure: in the first stage, a bone cement spacer is implanted into the bone defect area to induce the formation of a bioactive membrane rich in growth factors such as vascular endothelial growth factor (VEGF) and bone morphogenetic protein (BMP) [3]; in the second stage, autologous bone granules are implanted, utilizing the "bioreactor" function of the induced membrane to promote bone regeneration [4]. However, clinical practice has shown that issues such as the prolonged bone graft mineralization period following IMT surgery remain difficult to resolve [5]. The prolonged mineralization process means that patients have to endure the inconveniences caused by fixation devices for an extended period. This not only severely affects patients' quality of life but may also lead to complications such as joint stiffness and muscle atrophy, hindering the recovery of limb function. Meanwhile, the long treatment duration also imposes a heavy financial burden on patients. Therefore, optimizing the microenvironment of the induced membrane and regulating the death pathways of key cells represent crucial directions for enhancing the osteogenic efficacy of IMT.

Ferroptosis, a form of iron-dependent non-apoptotic cell death discovered in 2012, is characterized by intracellular iron overload, accumulation of lipid peroxides, and inactivation of glutathione peroxidase 4 (GPX4) [6]. Unlike traditional cell death modalities such as apoptosis and necrosis, ferroptosis generates reactive oxygen species (ROS) through Fenton reactions, directly damaging the phospholipid bilayer of cell membranes and leading to cellular disintegration [7]. In bone metabolism, osteoblasts, as the primary functional cells responsible for bone formation, have their survival and differentiation status directly determining bone regeneration efficiency [8, 9]. Accordingly, inhibiting the occurrence of ferroptosis in osteoblasts and reducing lipid peroxidation can protect osteoblast function and promote their bone formation. Recent studies have confirmed that ferroptosis plays a significant role in osteoporosis, osteoarthritis, and bone defect repair [10–12]. However, existing research has predominantly focused on phenotypic inhibition of ferroptosis, with insufficient exploration of its upstream regulatory mechanisms (particularly the core transcription factors involved in redox balance) and a lack of specific interventions targeting ferroptosis in osteoblasts within the induced membrane microenvironment.

Nuclear factor erythroid 2-related factor 2 (NRF2) serves as a core transcription factor in cellular responses to oxidative stress. It regulates the expression of over 200 downstream antioxidant enzymes (such as HO-1, NQO1 and GCLC) and iron metabolism-related proteins (such as FPN1 and FTH1) by binding to antioxidant response elements (ARE) [13, 14]. In the regulation of ferroptosis, NRF2 exerts its effects through a dual mechanism: on one hand, NRF2 directly activates the transcription of GPX4, enhancing the cell's capacity to eliminate lipid peroxidation [15]; on the other hand, NRF2 upregulates the system Xc-/GSH axis, maintaining intracellular glutathione (GSH) levels and inhibiting the occurrence of ferroptosis [16].

*Drynaria roosii* Nakaike is an epiphytic fern species belonging to the genus Drynaria in the family Polypodiaceae. It is renowned for its kidney-tonifying and bone-strengthening properties [17] and is widely used in the treatment of various orthopedic diseases, including fractures, bone defects and osteonecrosis [18–20]. Total flavonoids of *Rhizoma drynariae* (TFRD), the extracts from the dried rhizome of the plant, have been developed into a marketed drug—Qianggu Capsules (Drug Approval Number: Z20030007). Modern research has demonstrated that TFRD possesses unique advantages in multi-target regulation of bone metabolism [17]. It can not only promote osteoblast differentiation by activating the Wnt/β-catenin signaling pathway, but also inhibit RANKL-mediated osteoclastogenesis [21, 22]. TFRD has shown promising effects in the fields of osteoporosis and bone defect repair, making it a hot natural product in bone tissue engineering research [21, 22]. However, whether TFRD can inhibit ferroptosis in osteoblasts by activating the NRF2 signaling pathway, thereby improving the bone regeneration efficacy of the IMT, has not been reported. To address this question, we established a rat femoral bone defect model combined with the IMT to verify, at multiple levels both in vitro and in vivo, the effects of TFRD on ferroptosis in osteoblasts, the NRF2 signaling pathway, and bone regeneration efficacy, as well as to elucidate its underlying molecular mechanisms.

## Materials and methods

### Animals and grouping

According to the random number table method, sixty SPF-grade male SD rats (aged 8–9 weeks) were divided into five groups: Model group, low-dose TFRD (L-TFRD) group, high-dose TFRD (H-TFRD) group, positive control (DMF) group and sham operation (Sham) group, with 12 rats in each group. Except for the Sham group, rats in the remaining groups underwent the establishment of an IMT model on the right femur. After the second-stage surgery, rats in the L-TFRD and H-TFRD groups were gavaged with Qianggu Capsules (Beijing Qihuang Pharmaceutical Co., Ltd.; Approval Number: Z20030007; active ingredient: TFRD). Based on body weight calculations, the doses for L-TFRD and H-TFRD groups were 0.11 and 0.22 g/kg/d, respectively. Rats in the DMF group were gavaged with the NRF2 activator-DMF at a dose of 50 mg/kg/d. Rats in the Model group and Sham operation group were gavaged with an equal volume of normal saline to that of TFRD. Osteogenesis-related and ferroptosis-related indicators were detected 4 weeks and 8 weeks after drug administration, respectively. This study was conducted in accordance with internationally recognized principles (US guidelines, NIH publication #85–23, revised in 1985) for the use and care of laboratory animals. The animal experiments complied with the requirements of the Ethics Committee of Guangzhou University of Chinese Medicine (License Number: 20221202001).

### IMT model

The model was established in accordance with previously reported methods in the literature [23]. After anesthesia, the rats were secured on the operating table, and the surgical area was shaved and disinfected. Phase 1: A longitudinal incision, approximately 3 cm in length, was made on the lateral side of the distal femur. The muscle tissues were dissected layer by layer to expose the right femur. A customized six-hole miniplate was placed on the anterolateral aspect of the femur. After fixation with two self-tapping cortical screws at both the proximal and distal ends, a 4 mm segmental osteotomy was performed at the central part of the femoral shaft using a wire saw. Subsequently, a prefabricated cylindrical polymethylmethacrylate (PMMA) spacer was filled into the remaining bone defect area. The incision was then irrigated with normal saline and sutured layer by layer with silk threads. After 4–6 weeks, when the induced membrane had formed around the bone cement, the second-stage surgery was performed. Phase 2: The skin and the induced membrane were incised longitudinally along the previous surgical incision. The PMMA spacer was removed. Part of the rat's tailbone was harvested, cut into small bone particles, and implanted into the induced membrane. Finally, the induced membrane, fascia and skin were sutured sequentially. After the operation, penicillin (40,000 units per day) was administered via intramuscular injection to prevent infection.

### Micro-CT

The right femur of each rat was promptly extracted, with the surrounding muscle tissues carefully removed, and then placed into a fixed container. The container holding the femur was positioned inside a Micro-CT device (BRUKER, Skyscan 1172). Centering on the bone graft area, appropriate scanning parameters were set up, and a comprehensive scan of the femur was conducted to obtain high-resolution two-dimensional image data. After the scan was completed, three-dimensional reconstruction of the images was performed, and bone volume fraction (BV/TV) of femur was analyzed to evaluate bone structure and bone mass.

### HE, Safranin O-fast green (SO-FG) and Prussian blue staining

The right femur of the rat was harvested and subjected to fixation, decalcification, dehydration, clearing, and paraffin embedding before being sliced into thin sections. The sections were sequentially dewaxed in xylene and then dehydrated through a gradient of alcohol concentrations. The nuclei were stained with hematoxylin solution, followed by rinsing with running water. Subsequently, eosin, SO-FG, and Prussian blue staining solutions were added. After mounting with neutral balsam, the morphological and structural characteristics of the bone tissue were observed under a microscope.

### Immunohistochemistry

Paraffin sections of rat femurs were obtained, followed by dewaxing and hydration, and then antigen retrieval was performed. Endogenous peroxidase was blocked with 3% hydrogen peroxide, and non-specific sites were sealed with normal serum. Primary antibodies were added and incubated overnight. After rinsing with PBS, secondary antibodies were applied at room temperature. The sections were developed using DAB chromogenic solution, and the nuclei were counterstained with hematoxylin. After dehydration and clearing, the sections were mounted and observed under a microscope to assess the expression and localization of the target proteins. The area of the positive regions for the target protein in the images was analyzed using Image J software. The information on the primary antibodies were as follows: anti-BMP-2 antibody (1:100, Bioworld, United States, BS6815) and anti-RUNX2 antibody (1:400, Servicebio, China, GB115631).

### TUNEL assay

Paraffin sections of rat femurs were obtained and subjected to routine dewaxing and hydration. Antigen retrieval was performed using Proteinase K, followed by rinsing with PBS. TUNEL reaction solution was added dropwise, and the sections were incubated in a humidified chamber in the dark to allow the binding of the marker to fragmented DNA. After rinsing with PBS, DAB chromogenic solution was added for color development. The nuclei were counterstained with hematoxylin for approximately 3 min, followed by dehydration with absolute ethanol and clearing with xylene. After mounting, apoptotic cells, which appeared brownish-yellow, were observed under a fluorescence microscope to detect cellular apoptosis in the bone tissue.

### Extraction and culture of osteoblasts

The calvariae were harvested from neonatal SD rats, and the soft tissues were meticulously removed before the calvariae were minced. The cells were then isolated through alternating digestion with trypsin and collagenase. The obtained cell suspension was centrifuged, and the supernatant was discarded. The cells were resuspended in DMEM supplemented with 10% fetal bovine serum and 1% penicillin–streptomycin double antibiotic solution, and then seeded into culture flasks. The flasks were placed in a 37℃, 5% CO₂ incubator for cultivation. When the cells reached 80–90% confluence, they were subcultured at a ratio of 1:2 to obtain rat osteoblasts for subsequent experiments.

### Transfection of si-NRF2

Three small interfering RNA sequences targeting NRF2 (si-NRF2 #1, si-NRF2 #2, si-NRF2 #3) and a negative control sequence (si-NRF2 NC) were synthesized separately by GeneCreate (China). The osteoblasts were seeded into culture plates, and when osteoblasts confluence reached 60%, transfection was performed using Lipofectamine^™^ 3000 reagent (Invitrogen, USA). Twenty-four hours after transfection, the mRNA and protein levels of NRF2 were detected by qRT-PCR and Western blot, respectively, to evaluate the interfering effects of si-NRF2 sequences. The sequence information for each si-NRF2 was presented in Table 1.

**Table 1:** The sequences for transfected si-RNA and negative control (NC)

Gene	Sense (5′−3′)	Antisense (5′−3′)
si-NRF2 #1	GGAUGAAGAGACCGGAGAAUU	UUCUCCGGUCUCUUCAUCCAG
si-NRF2 #2	CGAGUUACAGUGUCUUAAUAC	AUUAAGACACUGUAACUCGGG
si-NRF2 #3	CCCGAGUUACAGUGUCUUAAU	UAAGACACUGUAACUCGGGAA
si-NRF2 NC	UUCUCCGAACGUGUCACGUTT	ACGUGACACGUUCGGAGAATT

### Screening for the optimal dosage of TFRD

The TFRD were dissolved in DMSO and stored at − 80℃. For use, the solution was diluted with DMEM to the desired concentrations. Osteoblasts were seeded into 96-well plates and cultured for 0, 24, 48 and 72 h in media containing varying concentrations of TFRD (0, 25, 50, 75, 100 and 125 μg/ml). Cell activity was then assessed using the CCK-8 assay. Based on the activity values, the optimal dosage of TFRD was determined.

### Grouping of osteoblasts

Osteoblasts were divided into six groups: Control group, Erastin group, Erastin + TFRD group, Erastin + DMF group, Erastin + si-NRF2 group and Erastin + si-NRF2 + TFRD group. After the completion of si-NRF2 transfection, 10 μmol/L Erastin was added to the DMEM in both the Erastin + si-NRF2 group and the Erastin group. Cells in the Erastin + TFRD group and the Erastin + si-NRF2 + TFRD were cultured in DMEM containing both Erastin and TFRD (50 μg/ml), while cells in the Erastin + DMF group were cultured in DMEM containing both Erastin and DMF (50 μM). The Control group was cultured with normal DMEM without any intervention.

### Cell viability

Rat osteoblasts were seeded into 96-well plates. After treatment for 0, 24, 48 and 72 h respectively, CCK-8 reagent (Servicebio, China, G4103) was added to each well. After incubation at 37℃ for 2 h, the optical density (OD) was measured at a wavelength of 450 nm using a microplate reader to analyze the effects of the treatment factors on osteoblast viability.

### Alkaline phosphatase (ALP) and Alizarin red s (ARS) staining

After treating osteoblasts with different methods for 24 h, the original culture medium was replaced with osteogenic induction medium. Following 2–3 weeks of induction culture, the cells were fixed with a 4% paraformaldehyde solution. After aspirating the fixative, an appropriate amount of ALP or ARS solution was added, and the cells were stained at room temperature for 30 min. The staining solution was then discarded, and the cells were thoroughly rinsed with PBS to remove unbound dye. The mineralization capacity of the osteoblasts was observed under an inverted microscope. Typical ALP staining appears purple, while mineralized nodules exhibit an orange-red color.

For the quantification of mineralization, ARS stain was dissolved in 10% cetylpyridinium chloride (Meilun Biological Technology, China) for one hour, then the solution was collected and distributed on a 96-well plate, and the OD value was measured at a 510 nm using a microplate reader.

### Measurement of total iron, MDA and GSH

After tissue homogenization or cell lysis, the supernatant was collected by centrifugation and used as the sample to be tested. The concentration of total iron in the samples was detected using a Total Iron Assay Kit (Servicebio, G4301, China); the content of malondialdehyde (MDA) in the samples was measured using an MDA Detection Kit (Servicebio, G4300, China); and the content of glutathione (GSH) in the samples was determined using a Total Glutathione Assay Kit (Beyotime, S0052, China).

### ELISA

The supernatant, collected after centrifugation of tissue homogenates or cell lysates, was used to measure 4-hydroxynonenal (4-HNE) levels according to the instructions of the commercial kit (Sangon, D751041, China).

### ROS assay

After treating osteoblasts with different therapeutic interventions for 24 h, the culture medium was aspirated and discarded. Then, DCFH-DA working solution was added, and the cells were incubated in the dark for 30 min to allow the probe to enter the cells and be hydrolyzed by esterases into DCFH. Intracellular ROS could oxidize the non-fluorescent DCFH into fluorescent DCF. Subsequently, cells were washed with PBS to remove the probes that had not entered the cells, and the fluorescence intensity of ROS was detected using a fluorescence microscope.

### Transmission electron microscopy (TEM)

For bone tissue, fresh bone specimens were sectioned into 2–3 mm thin slices and decalcified using EDTA decalcifying solution. For cells, the samples were first collected by centrifugation. The specimens were then fixed with 1% osmium tetroxide in the dark for 2 h. After rinsing with PBS, the samples were dehydrated using a gradient of ethanol and acetone, followed by embedding in an embedding medium. Ultrathin Sects. (50–70 nm) were cut using an ultramicrotome and stained with a combination of uranyl acetate and lead citrate to enhance contrast. Finally, the ultrastructure of both cells and bone tissue was observed under a TEM.

### qRT-PCR

After treating with different therapeutic interventions, total RNA was extracted from both the cells and bone tissue, ensuring the quality and purity of the RNA. Subsequently, the RNA was reverse-transcribed into cDNA using reverse transcriptase. After designing specific primers (Table 2), qPCR amplification was performed using cDNA as the template, with 2 × SYBR Green fluorescent dye added to the reaction system. Fluorescent signals were monitored using a real-time fluorescence quantitative PCR instrument, and Ct values were exported. Using the housekeeping gene GAPDH as a reference, the expression levels of target genes were calculated using the 2^−ΔΔCt^ method.

**Table 2:** Prime sequences for qRT-PCR

Gene	Forward (5′−3′)	Reverse (5′−3′)
*Nrf2*	GCTGAACTCCTTAGACTCAAATCCC	TGCTGCCACCGCCACTG
*Gpx4*	ATGCCCGATACGCCGAGTG	ATTTCTTGATTACTTCCTGGCTCCTG
*Ho-1*	GGCACTGCTGACAGAGGAACAC	CCACGGTCGCCAACAGGAAAC
*Slc7a11*	CATCATCATCGGCACCGTCATC	TCCACAGGCAGACCAGAACAC
*Bmp-2*	AAAGCGTCAAGCCAAACACAAAC	ACATCACTGAAGTCCACATACAAAGG
*Runx2*	CATTCCACCACGCCGCTGTC	GCACCTGCCTGGCTCTCCTTAC
*Gapdh*	GGCAAGTTCAACGGCACAGTC	TCGCTCCTGGAAGATGGTGATG

### Western blot

Cells or bone tissue samples were collected, washed with pre-chilled PBS, and then lysed on ice with lysis buffer. After centrifugation, the supernatant was collected. Following protein concentration determination using the BCA method, proteins were separated by SDS-PAGE electrophoresis and transferred onto PVDF membranes. Non-specific binding sites were blocked with 5% skim milk. Diluted primary antibodies were added, and the membranes were incubated overnight at 4 ℃. After washing with TBST, secondary antibodies were added, and the membranes were incubated at room temperature for 1 h. Following another round of washing, the membranes were developed using ECL chemiluminescent reagents, and the target protein bands were detected using a chemiluminescence imaging system to analyze their expression levels.

### Immunofluorescence

After treating osteoblasts with different methods for 24 h, the cells were first fixed with 4% paraformaldehyde. Then, a membrane-permeabilizing working solution was added to increase the permeability of the cell membrane, and 5% BSA was used to block non-specific binding sites. Subsequently, the primary antibody (NRF2, 1:500, Beyotime, China) was added and incubated overnight at 4 ℃. After washing with PBST, a fluorescent secondary antibody was added and incubated for 1 h at room temperature in the dark. Following nuclear staining with DAPI, the slides were mounted with an anti-fade mounting medium. Finally, the fluorescence intensity of NRF2 was observed using a laser scanning confocal microscope.

### Analysis of TFRD and TFRD-containing serum by UHPLC-Q-Orbitrap HRMS

A randomization process was employed to divide the SD rats into two separate groups (n = 5, each group): TFRD-containing serum group and blank serum group. Rats in the TFRD-containing serum group were administered TFRD by gavage at a dose of 220 mg/kg twice daily for three consecutive days. Rats in the blank serum group were given an equal volume of normal saline (as that of TFRD) by gavage. Two hours after the last administration, blood was collected from the abdominal aorta of the rats, centrifuged at 3000 r/min for 15 min, and the supernatant was taken to obtain TFRD-containing serum and blank serum, respectively. The sera samples were preserved at − 80 °C to facilitate their utilization in subsequent experiments.

Methanol and grinding beads were added to both the TFRD solution and the TFRD-containing serum to extract the chemical constituents present within them. After centrifuging for 10 min, the supernatant was collected for analysis. A complete liquid chromatography-mass spectrometry (LC–MS) system was established by coupling a Q-Orbitrap high-resolution mass spectrometer with an UltiMate 3000 RS liquid chromatograph. Appropriate chromatographic conditions (Supplementary Table 1) and mass spectrometry conditions (Supplementary Table 2) were selected. The processed TFRD extract was injected into the liquid chromatography system for chromatographic separation. The separated chemical components were then subjected to mass detection and qualitative analysis using the Q-Orbitrap high-resolution mass spectrometer. The collected data was initially processed using CD 3.3 software (Compound Discoverer 3.3, Thermo Fisher), and the information was compared with the mzCloud database to tentatively identify the chemical components in TFRD and TFRD-containing serum.

### Molecular docking

Compound structures for components of TFRD-containing serum were obtained from PubChem database, and these SDF files were converted to PDB files using Open Babel 2.3.2. The receptor protein (NRF2) was retrieved from UniProt database and modified using AutoDockTools software to remove water molecules, add hydrogen atoms, and balance charges, before being saved in PDBQT format. With Naringin and Nicotiflorin serving as ligands, molecular docking of receptor protein with ligands was performed using AutoDock Vina 1.1.2. PyMOL was employed to generate visual representations of the docking results.

### Molecular dynamics simulation (MDS)

The MDS was performed using GROMACS software to investigate the interactions between Naringin and NRF2, as well as between Nicotiflorin and NRF2. The AMBER force field was selected for the force field description of proteins, and the GAFF force field was selected for the force field description of short peptide molecules. The water model was set to the TIP3P water model. The system temperature was set to a vacuum environment at 300 k, and simulation time was 100 ns. First, the constructed simulation system was subjected to energy balance in an absolute vacuum environment, using the steepest gradient algorithm for calculation; After energy balance, the MD process is completed. After the simulation, the RMSD, RMSF, Rg, SASA and Hydrogen-bond number were calculated to evaluate stability of complex and flexibility of protein residues.

### CETSA

After pre-treating osteoblasts with Naringin or DMSO (10 μM) for 4 h, cells were collected and resuspended in lysis buffer to ensure complete cell lysis. Following centrifugation at 12,000 rpm, the supernatant was collected, representing total protein solution. The total protein solution was then divided equally into 8 aliquots and heated for 3 min at gradient temperatures ranging from 37 to 72 °C. The total protein solution was centrifuged at 12,000 rpm again to remove the heat-denatured precipitated proteins. Finally, Western blot analysis was initiated to visualize and quantify the target proteins, and the CETSA solubility curve was plotted using GraphPad Prism.

### Statistical analysis

Statistical analysis was conducted using SPSS 22.0 software. The experimental data were initially presented as mean ± standard deviation (SD). First, normality and homogeneity of variance tests were performed on the data from each group to confirm that they met the prerequisites for one-way analysis of variance (ANOVA). One-Way ANOVA was employed to compare differences in anti-ferroptosis and osteogenesis-related indicators among different treatment groups (such as groups treated with different concentrations of TFRD, Sham groups, etc.). If the ANOVA results indicated significant differences (*P* < 0.05), the Least Significant Difference (LSD) method was further used for pairwise comparisons to clarify the specific differences between each group.

## Results

### TFRD promoted the growth of bone grafts within the induced membrane

**Fig. 1:**
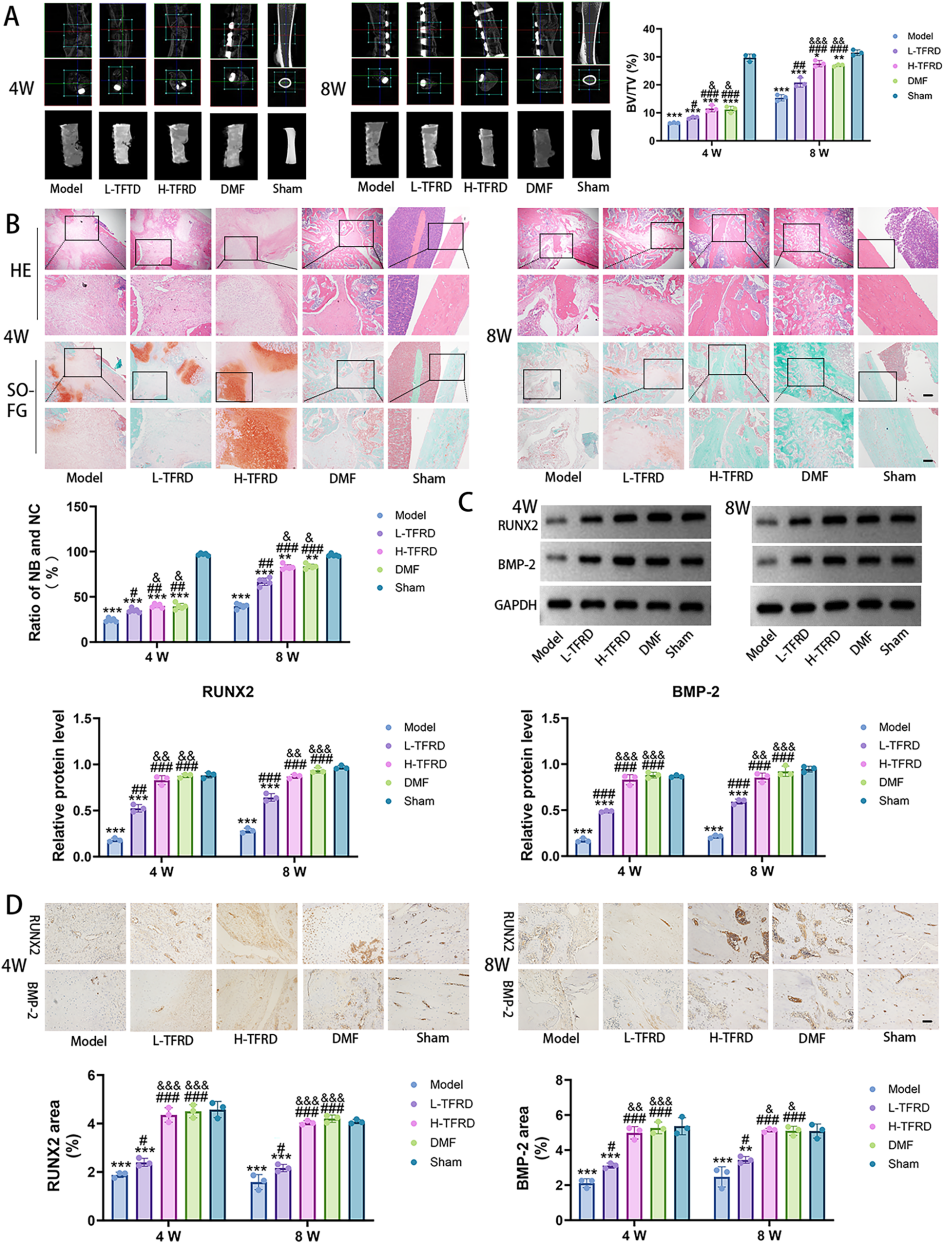
The influence of TFRD on the bone graft’s growth within the induced membrane. **A** was sagittal and transverse scan images as well as the 3D images of the right femurs from Micro-CT. The BV/TV was analyzed using CTAn software. **B** The histological characteristics of the bone graft were evaluated by HE and Safranin O-fast green (SO-FG) staining. Scale bar: 200 µm and 100 µm. **C** The protein level of RUNX2 and BMP-2 was detected by Western blot. **D** Immunohistochemistry result of RUNX2 and BMP-2. Scale bar: 50 µm. N = 3/group, biological replicates. Each value was presented as the mean ± SD. ^**^P < 0.01, ^***^P < 0.001 versus the Sham group;^#^*P* < 0.05, ^##^*P* < 0.01, ^###^*P* < 0.001 versus the Model group; ^&^*P* < 0.05, ^&&^*P* < 0.01, ^&&&^*P* < 0.001 versus the L-TFRD group

To investigate the effect of TFRD on osteogenesis in the IMT, initially, Micro-CT result revealed that the bone structure and bone volume fraction (BV/TV) in the Sham group were normal, with no significant abnormal changes. Compared with the Model group, the low- and high-dose groups of TFRD exhibited a significant increase in bone density within the bone graft region of the induced membrane, along with clearer and more continuous trabecular bone structures and higher BV/TV. Moreover, this improvement trend became more pronounced with increasing doses of TFRD. DMF also promoted bone graft growth, and the degree of improvement in BV/TV was similar to that observed in the H-TFRD group (Fig. 1A).

The results of HE and SO-FG staining provided strong support for the aforementioned findings at the histological level. After staining, it was found that in the bone graft area within the induced membrane of the Model group, there was a relatively large amount of fibrous tissue and less formation of new bone and new cartilage. In contrast, the L/H-TFRD and DMF groups exhibited a significantly increased amount of new bone and new cartilage, enhanced osteoblast activity, and increased bone matrix deposition. Moreover, the quality of new bone was higher in the H-TFRD and DMF groups (Fig. 1B).

Western blot and immunohistochemistry were further employed to detect the protein expression of key transcription factors involved in bone formation (RUNX2 and BMP-2) in the bone tissues of rats from each group. The results revealed that, compared to the Sham group, the protein expression of RUNX2 and BMP-2 in the Model group were significantly reduced, indicating that osteogenesis-related signaling pathways within the induced membrane in the Model group were suppressed. In contrast, L/H-TFRD groups exhibited significantly elevated expression of RUNX2 and BMP-2 compared to the Model group, with a gradual increase in expression corresponding to increasing doses. Furthermore, there were no significant differences in the protein levels of these osteogenic factors between H-TFRD and DMF groups (Fig. 1C, D). These findings suggested that both TFRD and DMF significantly promote the differentiation of osteoblasts and bone formation in the bone graft area.

### TFRD inhibited the occurrence of ferroptosis in the bone graft area of IMT

To explore the specific mechanism by which TFRD promotes osteogenesis in the IMT, we measured ferroptosis-related indicators such as iron ion and lipid peroxidation levels.

**Fig. 2:**
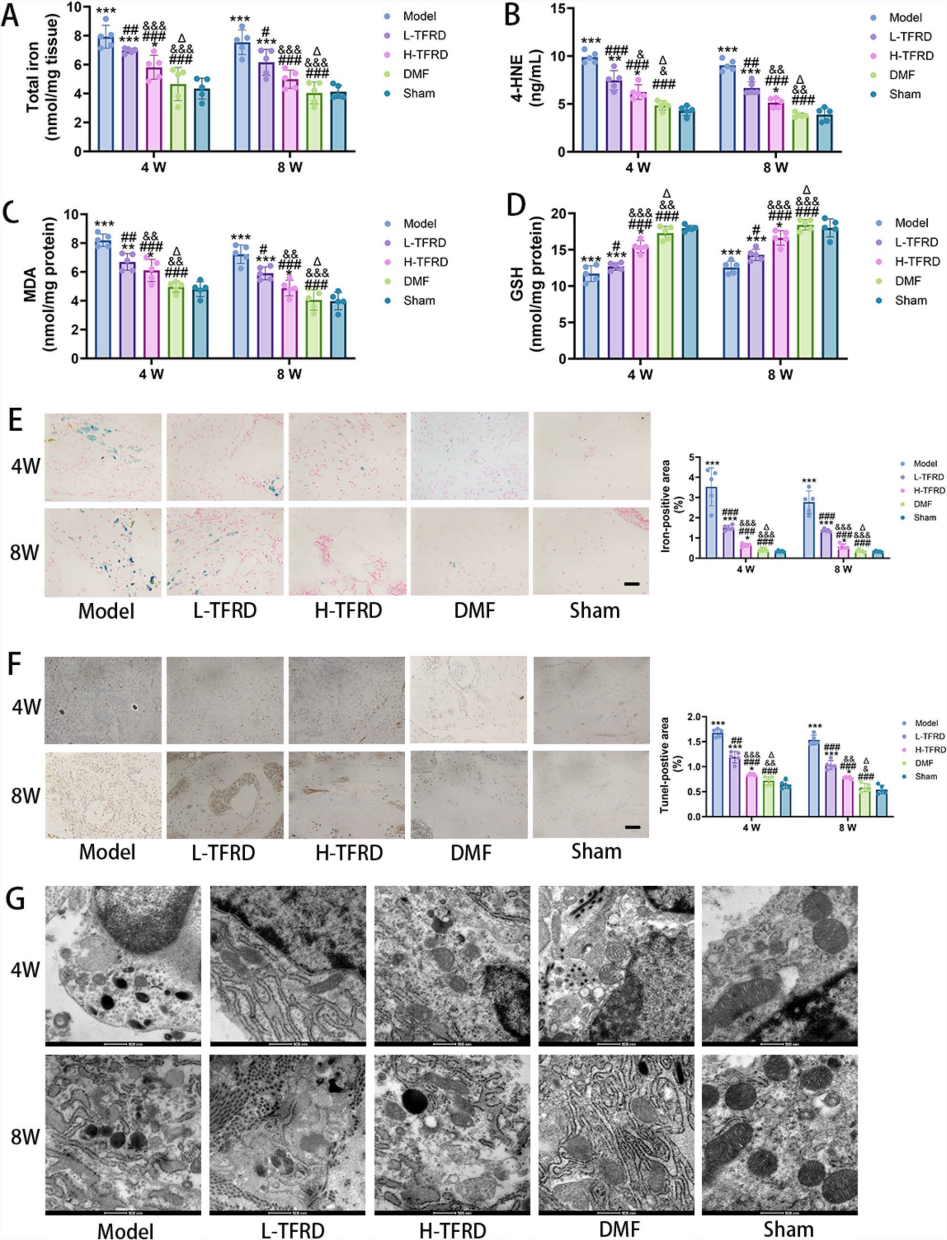
The effect of TFRD on indicators associated with ferroptosis in the bone graft area within the induced membrane. **A** The Total Iron Assay Kit was adopted to detect the total iron concentration in bones. **B** The content of 4-HNE in bones was measured using the ELISA kit. **C** The MDA Assay Kit was used to test the MDA content in bones. **D** The Total GSH Assay Kit was employed to detect the GSH content in bones. **E** Prussian blue staining was used to observe iron elements in bone tissue cells. Iron ions were blue, and cell nuclei were red. **F** TUNEL staining served as a method for identifying apoptotic cells. Apoptotic positive cell nuclei were stained brownish yellow, while normal cell nuclei were stained blue. **G** Ultrastructural characteristics of ferroptosis in bone tissue observed under transmission electron microscopy (TEM). N = 5/group, biological replicates. Each value was presented as the mean ± SD.^*^P < 0.05, ^**^P < 0.01, ^***^P < 0.001 versus the Sham group; ^#^*P* < 0.05, ^##^*P* < 0.01, ^###^*P* < 0.001 versus the Model group; ^&^*P* < 0.05, ^&&^*P* < 0.01, ^&&&^*P* < 0.001 versus the L-TFRD group. ^Δ^*P* < 0.05 versus the H-TFRD group

It is widely acknowledged that excessive iron accumulation is one of the important prerequisites for the occurrence of ferroptosis. The results of total iron detection showed that, compared with the Model group, the total iron content in the bone graft area of the induced membrane significantly decreased after TFRD and DMF interventions. Moreover, as the dose of TFRD increased, the decline in total iron content became more pronounced (Fig. 2A). This indicated that TFRD could effectively regulate iron metabolism in the bone graft area of IMT and reduce iron accumulation.

MDA and 4-HNE are iconic products of lipid peroxidation, and their contents can reflect the degree of intracellular oxidative stress and lipid peroxidation. Through detection, it was found that the contents of MDA and 4-HNE in the bone graft area of the induced membrane in the Model group were significantly higher than those in the Sham-operated group, indicating severe oxidative stress and lipid peroxidation damage in the Model group. After TFRD and DMF interventions, the contents of MDA and 4-HNE significantly decreased, with a greater reduction in the DMF group (Fig. 2B, C). GSH is an important antioxidant substance that plays a crucial role in maintaining cellular redox balance. The results of quantitative GSH detection showed that the GSH content in the bone graft area of the Model group was significantly lower than that in the Sham group, while TFRD could dose-dependently enhance the GSH content (Fig. 2D). This suggested that TFRD could improve the antioxidant capacity of cells in the bone graft area of the induced membrane and enhance the cells' resistance to oxidative damage such as ferroptosis. Additionally, the improvement of these indicators in the DMF group was more significant, and its inhibitory effect on ferroptosis was stronger than that of the H-TFRD group.

Prussian blue staining is a method for visually demonstrating iron deposition in tissues. The staining results showed that a large number of blue iron particles were deposited in the bone graft area of the Model group, while iron particle deposition significantly decreased in the TFRD intervention groups, especially in the H-TFRD group (Fig. 2E). This corroborated with the results of total iron detection and further confirmed that TFRD could reduce iron deposition in the bone graft area.

TUNEL staining is commonly used to detect cell apoptosis, and ferroptosis differs from cell apoptosis in terms of morphology and mechanism. The TUNEL result showed that there were a relatively large number of TUNEL-positive cells in the bone graft area of the Model group, indicating a certain degree of cell death. After TFRD treatment, the number of TUNEL-positive cells significantly decreased (Fig. 2F), suggesting that TFRD could reduce cell death in the bone graft area and protect cells from damage such as ferroptosis.

TEM is the gold standard for observing changes in cell ultrastructure. Through TEM observation, it was found that some cells in the bone graft area of the Model group exhibited typical ferroptosis characteristics, such as smaller mitochondria, increased membrane density, and reduced or even disappeared cristae. However, the degree of mitochondrial damage was alleviated in the low- and high-dose TFRD groups, with more pronounced improvement in the high-dose group. The mitochondrial morphology in the DMF group was close to normal, with the least severe damage (Fig. 2G). This provided strong evidence at the cellular ultrastructure level that TFRD inhibited the occurrence of ferroptosis in the bone graft area of the induced membrane.

Based on the comprehensive results above, we could observe that: TFRD had effectively inhibited the occurrence of ferroptosis in the bone graft area of the induced membrane through multiple pathways, such as regulating iron metabolism, alleviating oxidative stress and lipid peroxidation, and enhancing antioxidant capacity. However, the inhibitory effect of TFRD on ferroptosis was not as potent as that of DMF.

### TFRD upregulated the NRF2/ARE pathway factors in the bone graft area of IMT

**Fig. 3:**
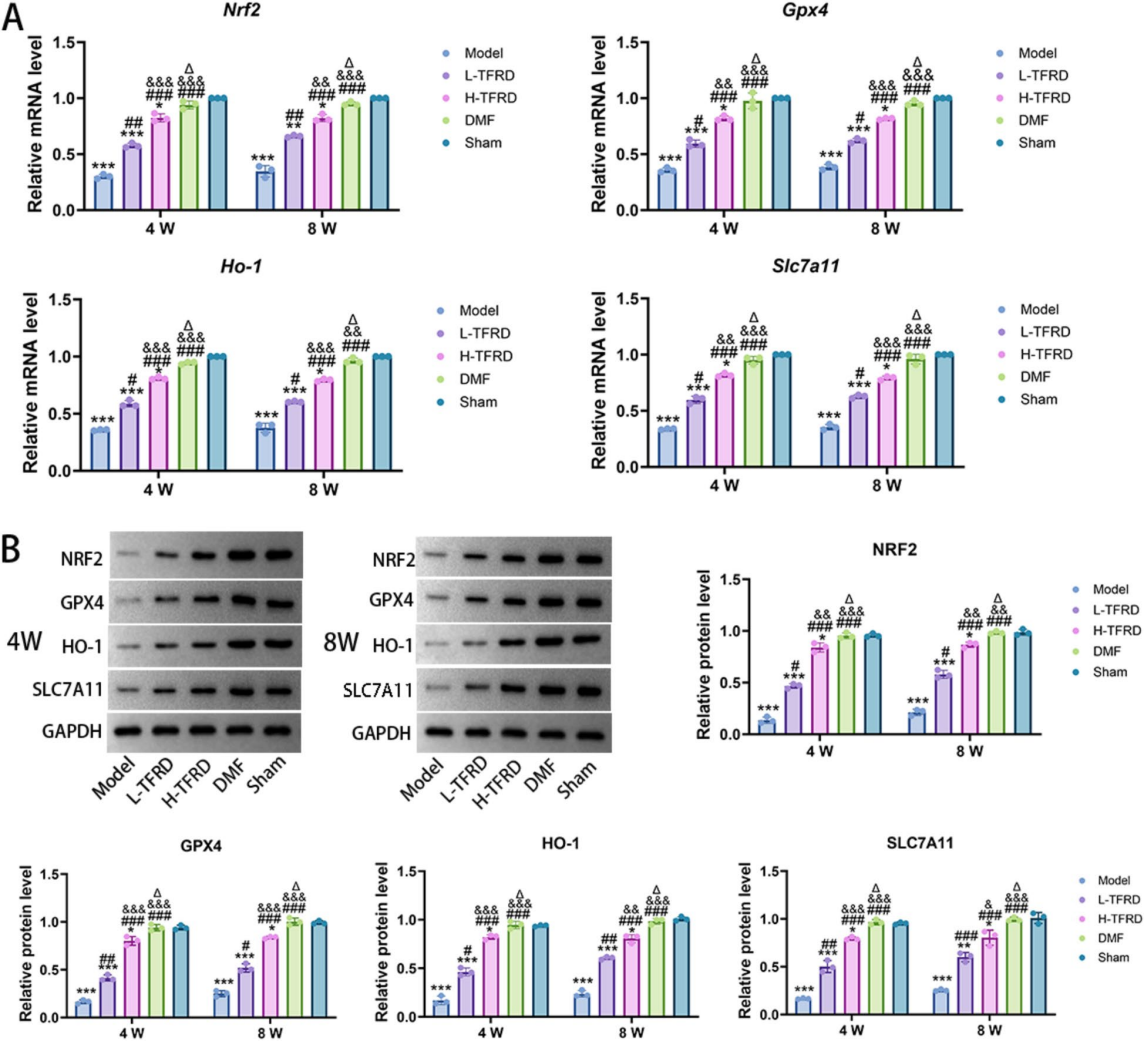
The impact of TFRD on NRF2/ARE pathway factors in the bone graft area of IMT. **A** The mRNA level of *Nrf2*, *Gpx4*, *Ho-1* and *Slc7a11* was detected by qRT-PCR. **B** The protein level of NRF2, GPX4, HO-1 and SLC7A11 was detected by Western blot. N = 3/group, biological replicates. Each value was presented as the mean ± SD. ^*^P < 0.05, ^**^P < 0.01, ^***^P < 0.001 versus the Sham group;^#^*P* < 0.05, ^##^*P* < 0.01, ^###^*P* < 0.001 versus the Model group; ^&^*P* < 0.05, ^&&^*P* < 0.01, ^&&&^*P* < 0.001 versus the L-TFRD group. ^Δ^*P* < 0.05 versus the H-TFRD group

The NRF2/ARE pathway plays a crucial regulatory role in the occurrence of ferroptosis. The results of qRT-PCR revealed that, in the bone graft area of the induced membrane, compared with the Model group, the mRNA expression of key factors (*Nrf2*, *Gpx4*, *Ho-1* and *Slc7a11*) in the NRF2/ARE pathway were significantly elevated in the TFRD and DMF treatment groups. Specifically, under low-dose TFRD intervention, there was a marked increase in the mRNA expression of NRF2 from its relatively low level in the Model group, accompanied by concurrent increases in the mRNA expressions of *Gpx4*, *Ho-1* and *Slc7a11*. This upregulation was statistically significant (*P* < 0.05 vs. Model). As the dose of TFRD increased, the mRNA expression of *Nrf2*, *Gpx4*, *Ho-1* and *Slc7a11* further increased (*P* < 0.05 vs. L-TFRD), although these levels remained lower than those in the DMF group (*P* < 0.05 vs. DMF) (Fig. 3A). This indicated that TFRD could effectively activate the expression of factors related to the NRF2/ARE pathway at the transcriptional level, but its activation intensity was weaker than that of DMF. Western blot further validated the finding of qRT-PCR at the protein level, demonstrating that TFRD and DMF could upregulate the expression of NRF2/ARE pathway factors at the protein level (Fig. 3B).

### Transfection efficiency of si-NRF2

**Fig. 4:**
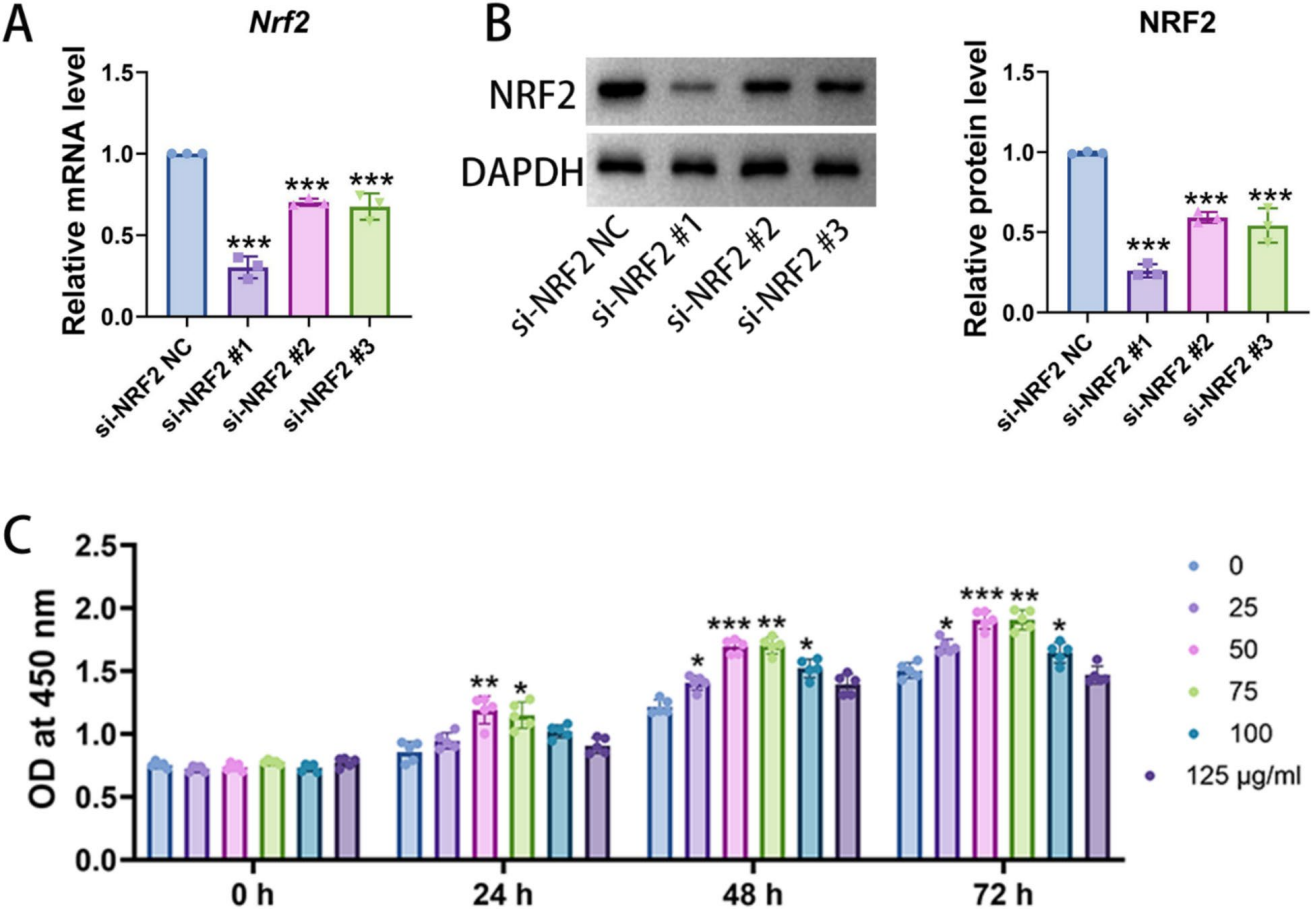
The transfection efficacy of si-NRF2 and screening for the optimal dose of TFRD. **A** qRT-PCR was utilized to validate the silencing effect of si- NRF2 #1, #2 and #3. **B** Western blot was used to validate the silencing effect of si-NRF2 #1, #2 and #3. N = 5/group, biological replicates. Each value was presented as the mean ± SD. ^***^P < 0.001 versus the si-NRF2 NC. **C** Osteoblast viability was assessed using the CCK-8 method at 24, 48 and 72 h. N = 5/group, biological replicates. Each value was presented as the mean ± SD. ^*^P < 0.05, ^**^P < 0.01, ^***^P < 0.001 versus the 0 μg/ml group

After transfection was completed, the qRT-PCR result showed that compared with the si-NRF2 NC group, the mRNA level of NRF2 in the si-NRF2 #1, #2, and #3 groups were all significantly reduced. Among them, the decrease was most pronounced in the si-NRF2 #1 group (Fig. 4A). The Western blot result was consistent with the qRT-PCR, indicating that the protein expression of NRF2 in these three groups was also significantly lower than that in the si-NRF2 NC group, with the si-NRF2 #1 group showing the best inhibitory effect (Fig. 4B). These findings suggest that the designed siRNAs can effectively interfere with the expression of NRF2 in osteoblasts.

### TFRD promoted the proliferative activity of osteoblasts under Erastin intervention

In the in vitro study, we conducted a screening for the optimal dose of TFRD. After culturing osteoblasts with different concentrations of TFRD for 0, 24, 48 and 72 h, the cell activity was measured using the CCK-8 method. The result showed that the effects of different concentrations of TFRD on cell proliferation varied. By synthesizing data from all stages, it was determined that the cell activity was optimal at concentrations of 50 and 75 μg/ml (Fig. 4C). Therefore, 50 μg/ml was selected as the optimal dose of TFRD for subsequent experiments.

**Fig. 5:**
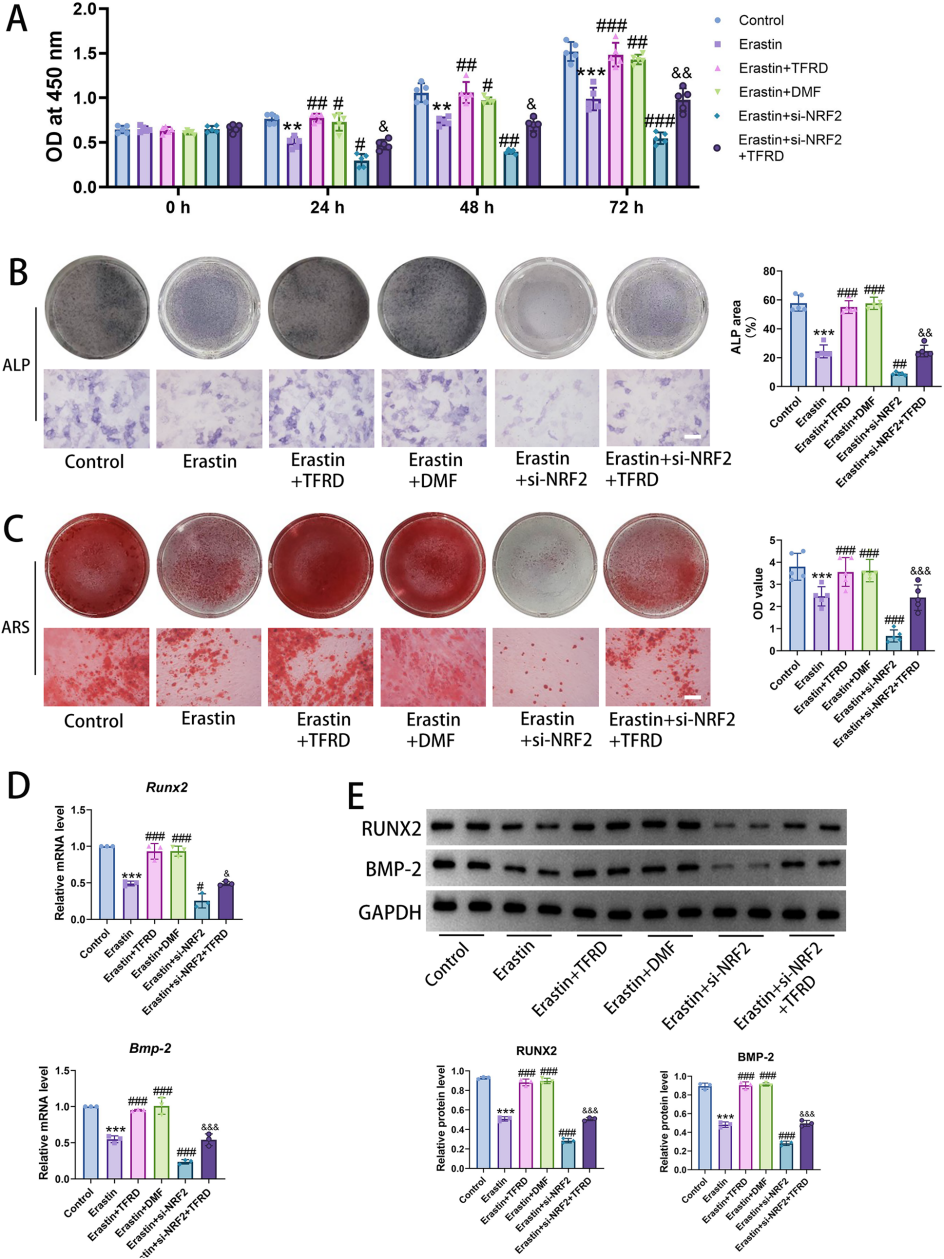
The effect of TFRD on the activity and mineralization function of osteoblasts induced by Erastin. **A** The effect of TFRD on the viability of osteoblasts induced by Erastin was assessed using the CCK-8 assay. N = 5/group, biological replicates. **B** ALP staining was used to detect the formation of ALP in osteoblasts. Scale bar: 50 µm. N = 5/group, biological replicates. **C** ARS staining was performed to detect mineralized nodules in osteoblasts. Scale bar: 50 µm. N = 5/group, biological replicates. **D** The mRNA level of osteogenic factors (*Runx2* and *Bmp-2*) in osteoblasts was detected by qRT-PCR. N = 5/group, biological replicates. **E** The protein level of osteogenic factors (RUNX2 and BMP-2) in osteoblasts was detected by Western blot. N = 5/group, biological replicates. Each value was presented as the mean ± SD. ^**^P < 0.01 versus the Control group; ^***^P < 0.001 versus the Control group; ^#^*P* < 0.05, ^##^*P* < 0.01, ^###^*P* < 0.001 versus the Erastin group;^&^*P* < 0.05, ^&&^*P* < 0.01, ^&&&^*P* < 0.001 versus the Erastin + si-NRF2 group

Subsequently, we examined the proliferative activity of osteoblasts under Erastin intervention at 24, 48, and 72 h. The CCK-8 result demonstrated that cells in the Control group exhibited stable proliferation at all time points, with OD value increasing over time. In the Erastin group, due to Erastin-induced ferroptosis, cell viability was significantly suppressed, with the OD value at each time point being lower than that in the Control group (*P* < 0.05). In contrast, the OD value in the Erastin + si-NRF2 group was even lower than those in the Erastin group. After the addition of TFRD in the Erastin + TFRD group, cell viability was markedly improved, with OD value at 24, 48, and 72 h being significantly higher than that in the Erastin group (*P* < 0.05) and comparable to that in the Erastin + DMF group. As time progressed, the promotional effects of TFRD and DMF became more pronounced. Meanwhile, the OD value in the Erastin + si-NRF2 + TFRD group was significantly higher than that in the Erastin + si-NRF2 group (Fig. 5A), indicating that TFRD not only promotes the proliferative activity of osteoblasts under Erastin intervention in a time-dependent manner but also reverses the inhibitory effect of si-NRF2 on cell viability.

### TFRD promoted the mineralization of osteoblasts under Erastin intervention

To investigate the effect of TFRD on the mineralization of osteoblasts induced by Erastin, the results of ALP and ARS staining revealed that a large number of purple ALP deposits and orange-red mineralized nodules were observed in the Control group, with a dense distribution. In the Erastin group, the formation of ALP and mineralized nodules was significantly reduced, and their colors became lighter. Compared with the Erastin group, the area of ALP and the numbers of mineralized nodules in the Erastin + TFRD group and the Erastin + DMF group were notably increased, with deeper colors, while those in the Erastin + si-NRF2 group were significantly smaller. Meanwhile, there was no significant difference in the formation of ALP and mineralized nodules between the Erastin + DMF group and the Erastin + TFRD group (Fig. 5B, C).

qRT-PCR and Western blot were employed to detect the mRNA and protein expression of RUNX2 and BMP-2. Compared with the Control group, both the mRNA and protein levels of RUNX2 and BMP-2 were significantly reduced in the Erastin group. In the Erastin + TFRD group and the Erastin + DMF group, the mRNA and protein expressions of RUNX2 and BMP-2 were significantly upregulated compared to the Erastin group. Furthermore, the upregulation in the Erastin + TFRD group was similar to that in the Erastin + DMF group. The mRNA and protein levels of these two factors in the Erastin + si-NRF2 group were significantly downregulated compared to those in the Erastin group, whereas the levels of these two factors in the Erastin + si-NRF2 + TFRD group were higher than those in the Erastin + si-NRF2 group (Fig. 5D, E).

These findings indicated that TFRD could effectively reverse the inhibitory effect of Erastin and si-NRF2 on osteogenic differentiation of osteoblasts, enhancing their osteogenic capacity. Moreover, the osteogenic-promoting ability of TFRD was similar to that of DMF.

### TFRD mitigated Erastin-induced ferroptosis in osteoblasts

**Fig. 6:**
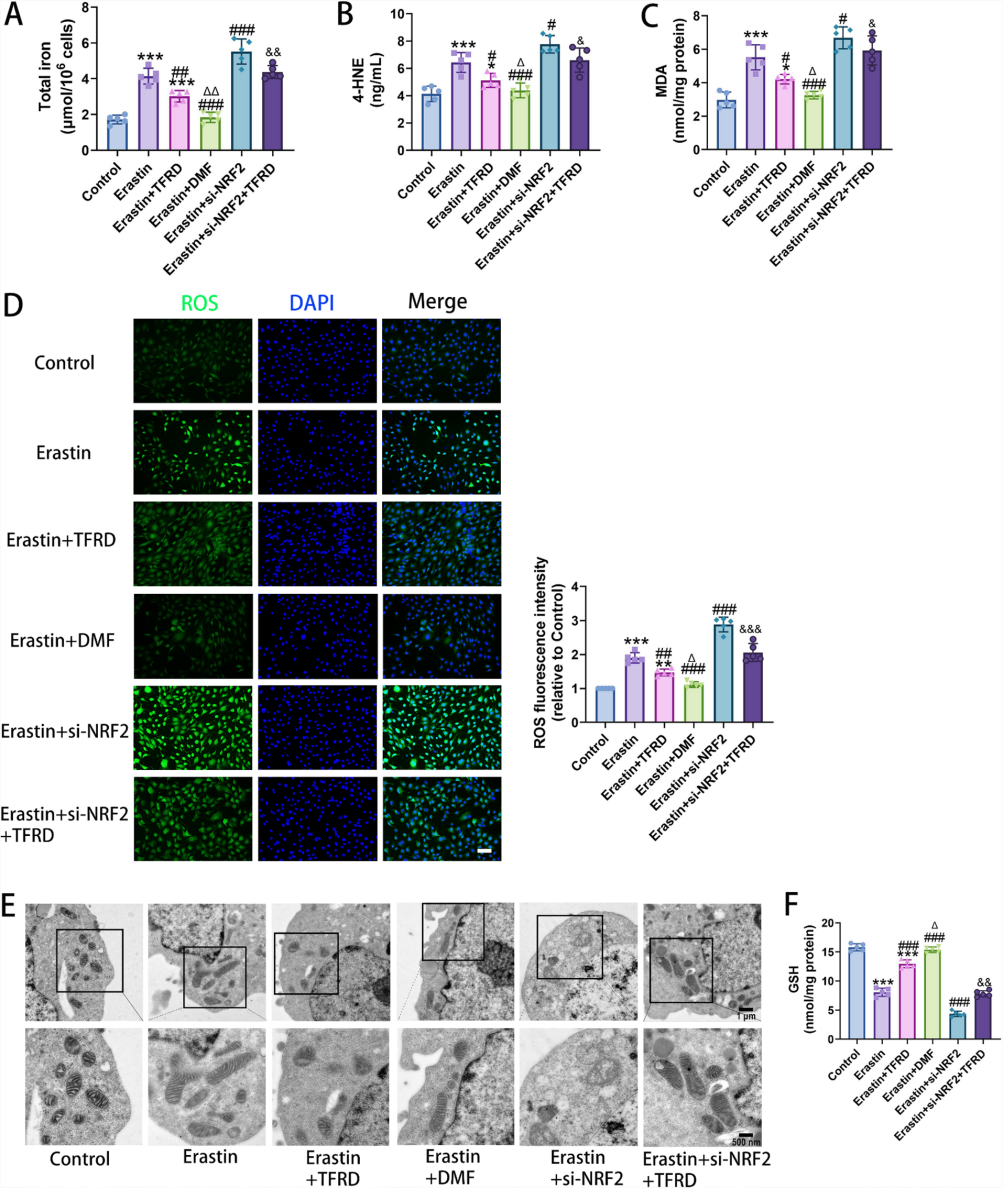
The impact of TFRD on ferroptosis of osteoblasts induced by Erastin. **A** The Total Iron Assay Kit was adopted to detect the total iron concentration in osteoblasts. N = 5/group, biological replicates. **B** The content of 4-HNE in osteoblasts was measured using the ELISA kit. N = 5/group, biological replicates. **C** The MDA Assay Kit was used to test the MDA content in osteoblasts. N = 5/group, biological replicates. **D** represented the fluorescence staining result of ROS in osteoblasts. Scale bar: 50 µm. N = 5/group, biological replicates. **E** The ferroptotic characteristics of Erastin-induced osteoblasts under the TEM. **F** The Total GSH Assay Kit was employed to detect the GSH content in osteoblasts. N = 5/group, biological replicates. Each value was presented as the mean ± SD.^*^P < 0.05, ^**^P < 0.01, ^***^P < 0.001 versus the Control group; ^#^*P* < 0.05, ^##^*P* < 0.01, ^###^*P* < 0.001 versus the Erastin group; ^&^*P* < 0.05, ^&&^*P* < 0.01, ^&&&^*P* < 0.001 versus the Erastin + si-NRF2 group; ^Δ^*P* < 0.05, ^ΔΔ^*P* < 0.01 versus the Erastin + TFRD group

In terms of iron metabolism-related indicators, compared with the Control group, total intracellular iron content in the Erastin group had significantly increased. Compared with the Erastin group, the total iron content was higher in the Erastin + si-NRF2 group, while it was lower in the Erastin + TFRD group and the Erastin + DMF group. Moreover, the total iron content in the Erastin + TFRD group had been lower than that in the Erastin + DMF group, indicating that TFRD could inhibit Erastin-induced intracellular iron accumulation, although its effect was weaker than that of DMF (Fig. 6A).

Detection of oxidative stress indicators had revealed a substantial rise in the levels of MDA and 4-HNE, typical products of lipid peroxidation, in the Erastin group and the Erastin + si-NRF2 group. The elevation of these substances had suggested an exacerbation of oxidative damage triggered by ferroptosis. Meanwhile, the GSH level, an important intracellular antioxidant, had significantly decreased. In the Erastin + TFRD group and the Erastin + DMF group, the levels of MDA and 4-HNE had decreased, while the GSH level had rebounded (Fig. 6B, C, F), indicating that both TFRD and DMF could alleviate oxidative stress damage caused by ferroptosis.

ROS fluorescence detection revealed that the intracellular ROS fluorescence intensity was significantly enhanced in both the Erastin group and the Erastin + si-NRF2 group, indicating a substantial generation of reactive oxygen species within the cells. Notably, the Erastin + si-NRF2 group exhibited the highest level of ROS production. In the Erastin + TFRD and Erastin + DMF groups, the ROS fluorescence intensity had diminished, suggesting that TFRD and DMF could reduce intracellular ROS levels in osteoblasts and suppress oxidative stress. However, the ability of TFRD to counteract oxidative stress had been lower than that of DMF (*P* < 0.05 vs. DMF group) (Fig. 6D).

TEM observation of intracellular ultrastructure had revealed that in the Erastin group, mitochondria in the cells had been significantly shrunken, with increased membrane density and reduced or even disappeared cristae, presenting typical mitochondrial morphological changes associated with ferroptosis. The mitochondrial morphological changes were even more pronounced in the Erastin + si-NRF2 group. In the Erastin + TFRD group and Erastin + DMF group, the degree of mitochondrial damage in cells had been alleviated, and their morphology had been closer to normal. The degree of mitochondrial damage in the Erastin + DMF group had been even lighter than that in the Erastin + TFRD group, with a more intact mitochondrial morphology (Fig. 6E).

Meanwhile, we observed that compared with the Erastin + si-NRF2 group, the Erastin + si-NRF2 + TFRD group exhibited significantly lower levels of total iron content, lipid peroxidation products (MDA and 4-HNE) and ROS concentration, along with higher GSH level and milder mitochondrial damage (Fig. 6A-E).

In summary, TFRD had effectively mitigated Erastin-induced ferroptosis in osteoblasts by regulating iron metabolism, alleviating oxidative stress, and protecting mitochondrial structure. However, its ability to counteract ferroptosis in osteoblasts had been weaker than that of DMF. In addition, TFRD could effectively reverse the ferroptosis-promoting effect of si-NRF2.

### TFRD upregulated the NRF2/ARE pathway in osteoblasts undergoing ferroptosis

**Fig. 7:**
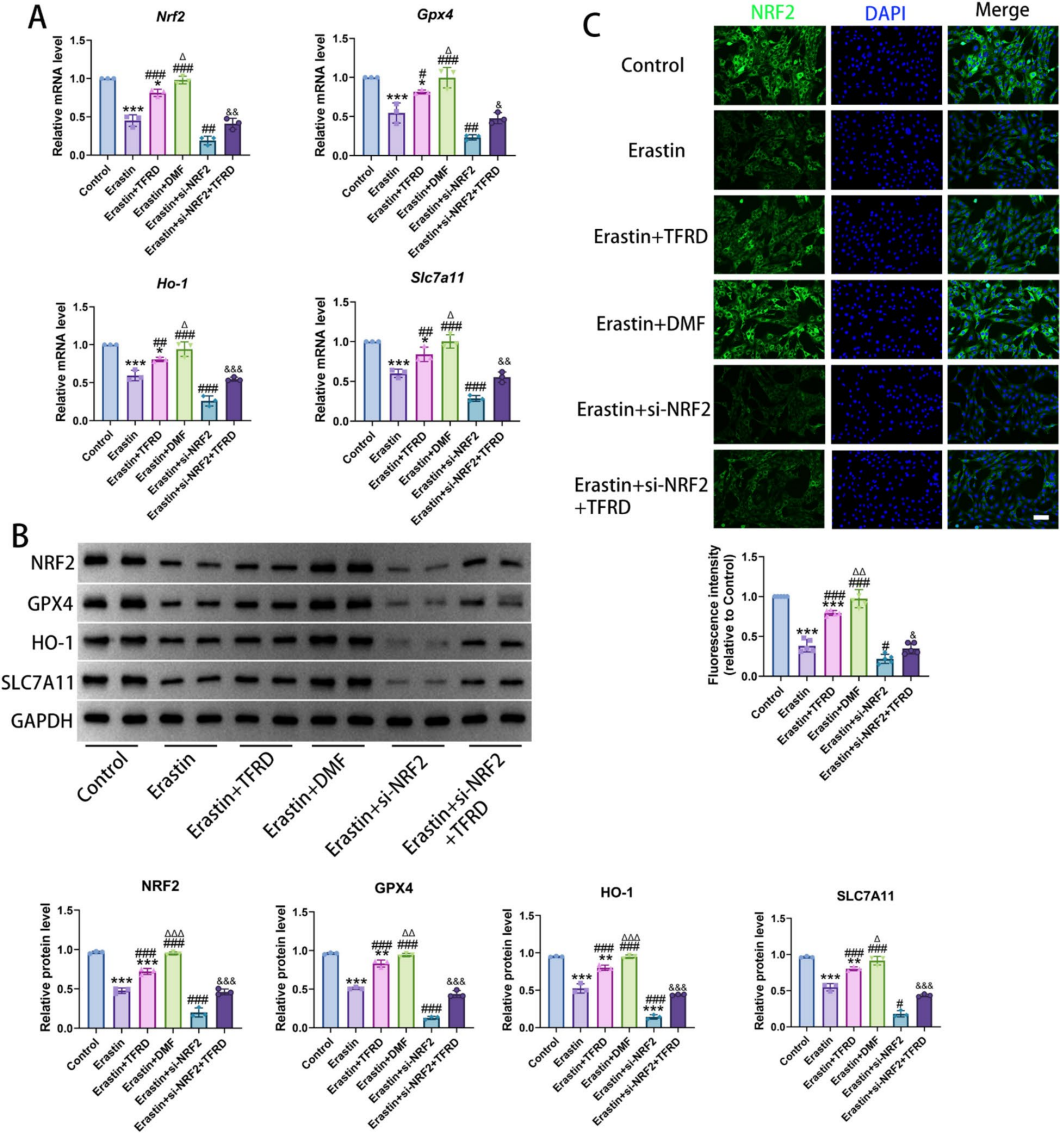
The impact of TFRD on NRF2/ARE pathway factors in osteoblasts induced by Erastin. **A** The mRNA level of *Nrf2*, *Gpx4*, *Ho-1* and *Slc7a11* was detected by qRT-PCR. **B** The protein level of NRF2, GPX4, HO-1 and SLC7A11 was detected by Western blot. **C** showed the immunofluorescence result of NRF2 in osteoblasts. Scale bar: 50 µm. N = 5/group, biological replicates. Each value was presented as the mean ± SD. ^*^P < 0.05, ^***^P < 0.001 versus the Control group; ^#^*P* < 0.05, ^##^*P* < 0.01, ^###^*P* < 0.001 versus the Erastin group;^&^*P* < 0.05, ^&&^*P* < 0.01, ^&&&^*P* < 0.001 versus the Erastin + si-NRF2 group; ^Δ^*P* < 0.05, ^ΔΔ^*P* < 0.01 versus the Erastin + TFRD group

To confirm the effect of TFRD on the NRF2/ARE pathway in osteoblasts undergoing ferroptosis in vitro, qRT-PCR and Western blot experiments were conducted. Compared with the Control group, both mRNA and protein levels of NRF2 were significantly decreased in the Erastin group. Compared with the Erastin group, the expression of NRF2 was significantly decreased in the Erastin + si-NRF2 group, while it was notably upregulated in the Erastin + TFRD group and the Erastin + DMF group. Meanwhile, the mRNA and protein levels of downstream antioxidant-related factors (GPX4, HO-1 and SLC7A11) were also significantly elevated in the Erastin + TFRD group and the Erastin + DMF group. Additionally, the level of factors related to the NRF2/ARE pathway in the Erastin + si-NRF2 + TFRD group were significantly higher than those in the Erastin + si-NRF2 group. This indicated that TFRD could activate the NRF2/ARE pathway and reverse the inhibitory effect of si-NRF2 on this pathway. Nevertheless, the ability of TFRD to upregulate factors related to the NRF2/ARE pathway was lower than that of DMF (Fig. 7A, B).

The NRF2 immunofluorescence further validated this result. In the Control group, NRF2 was mainly distributed in the cytoplasm. In the Erastin group, the fluorescence intensity of NRF2 in the cells was weakened, and it was still predominantly distributed in the cytoplasm. In contrast, in the Erastin + TFRD group, the fluorescence intensity of NRF2 was significantly enhanced, and its translocation into the nucleus was observed, suggesting that TFRD promoted the nuclear translocation of NRF2, thereby activating the expression of antioxidant genes mediated by ARE (Fig. 7C).

In conclusion, TFRD was able to upregulate the expression of NRF2 protein in osteoblasts undergoing ferroptosis, promote its nuclear translocation, activate the NRF2/ARE pathway, and enhance the cell's antioxidant capacity.

### Active constituents of TFRD and TFRD-containing serum

**Fig. 8:**
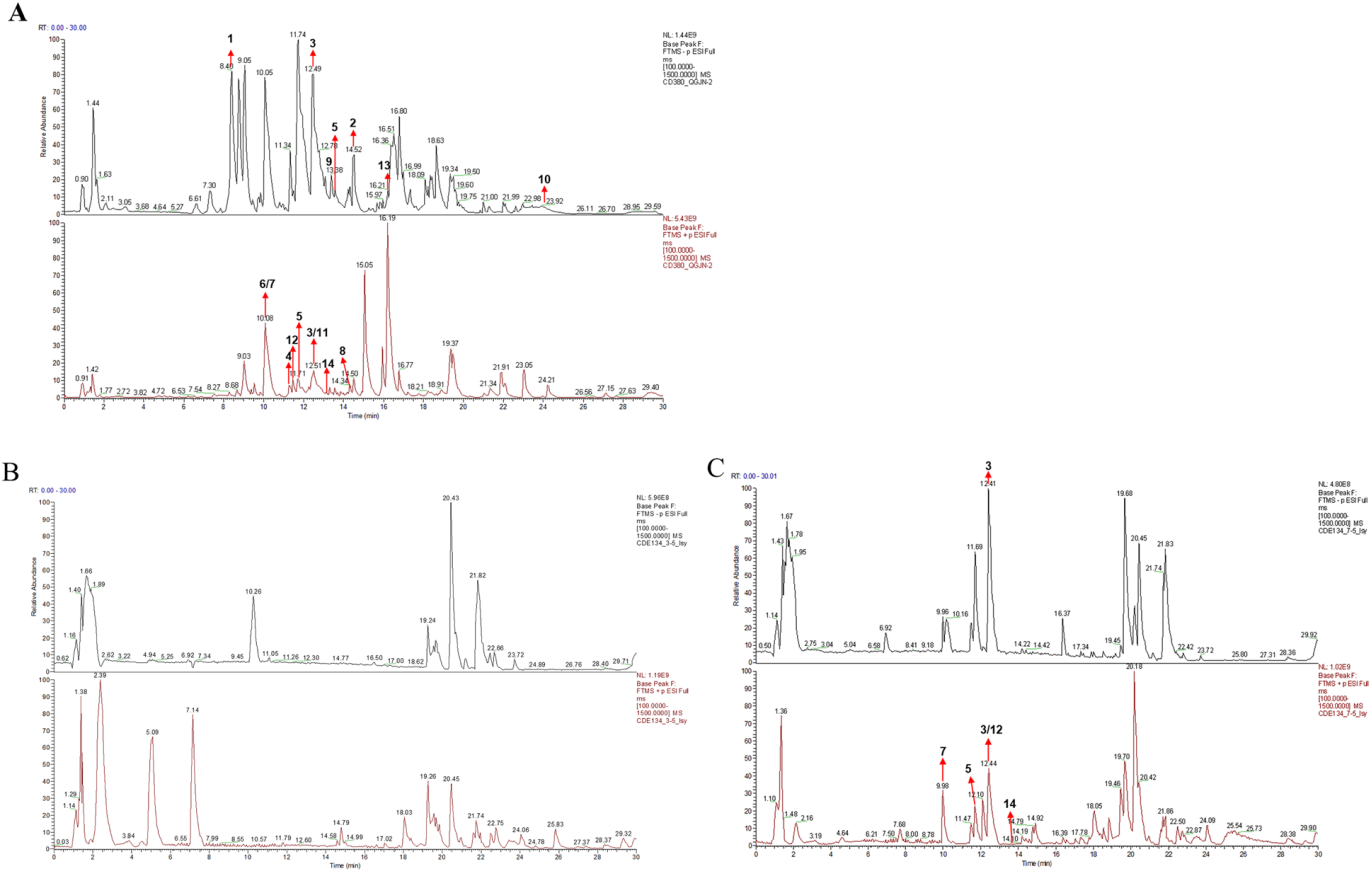
The total ion chromatogram of the UHPLC-Q-Orbitrap HRMS of TFRD **A**, blank serum **B** and TFRD serum **C** in negative and positive ion modes

The UHPLC-Q-Orbitrap HRMS identified 14 components of TFRD, including 9 flavonoids (Naringenin, Naringin, Neoeriocitrin, Eriodictyol, Kaempferol, Naringeninchalcone, Nicotiflorin, Luteolin and Kaempferol 7-O-glucoside, two coumarins (Esculin and Esculetin), two carboxylic acids (Azelaic acid and Stearic acid), and one phenolic compound (Gentisaldehyde.). Additionally, UHPLC-Q-Orbitrap HRMS analysis of TFRD-containing serum identified five major constituents: Naringin, Eriodictyol, Esculetin, Nicotiflorin and Kaempferol 7-O-glucoside. Figure 8 presented the total ion chromatograms of TFRD and TFRD-containing serum in both negative and positive ion modes during UHPLC-Q-Orbitrap HRMS. Supplementary Fig. 1–14 displayed the MS1 and MS2 mass spectra of these components. The chemical structures of these compounds were shown in Supplementary Fig. 15. Supplementary Table 3 listed the retention time and mass spectrometry fragment information of these compounds. The aforementioned analysis indicated that certain active components in TFRD were absorbed into the bloodstream, thereby exerting their therapeutic effects.

### The serum components of TFRD might directly bind to NRF2

**Fig. 9:**
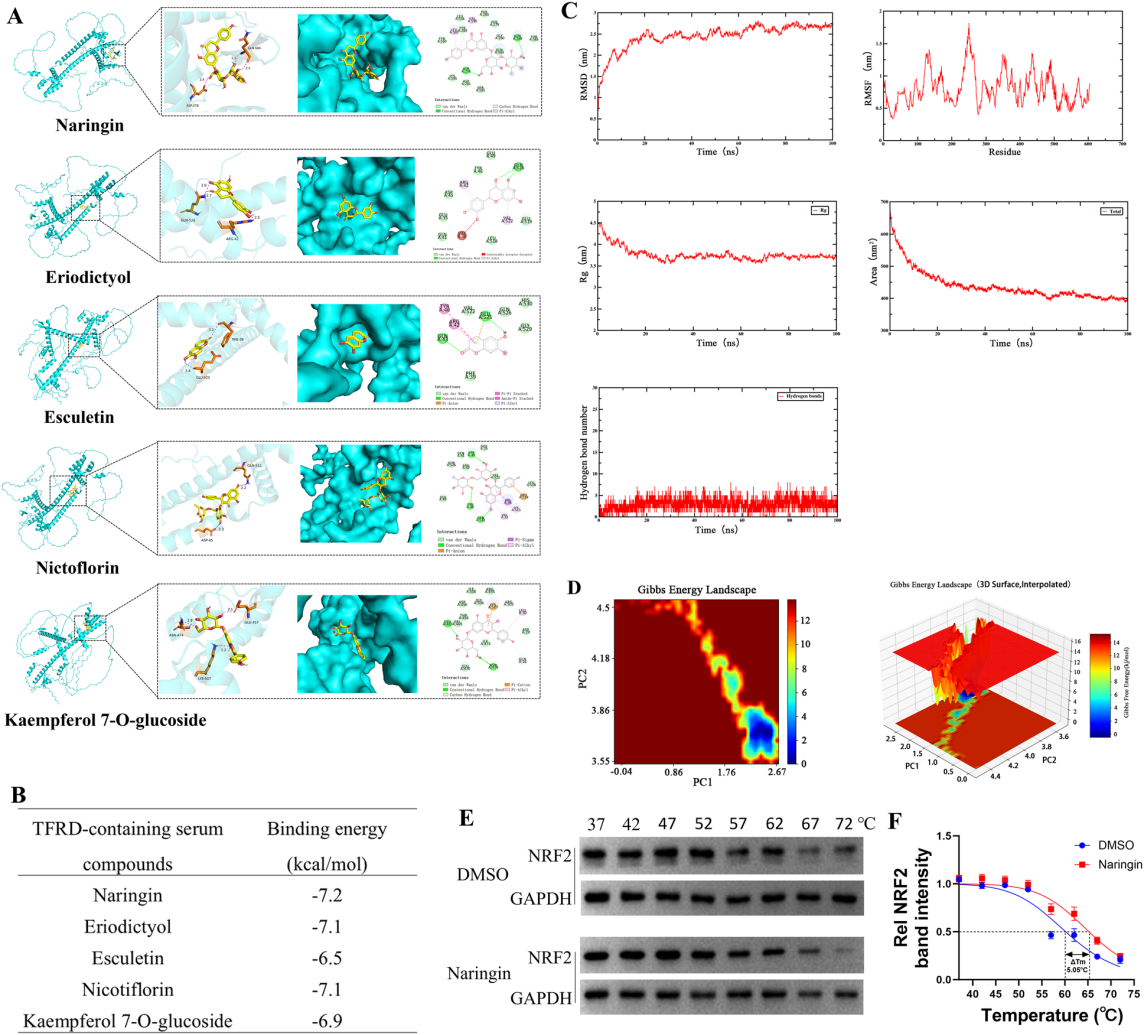
Interactions between TFRD-containing serum compounds and the NRF2. **A** Three-dimensional and two-dimensional molecular docking patterns of TFRD compounds with NRF2. **B** The binding energy between TFRD-containing serum components and NRF2. **C** Results of molecular dynamics simulation analysis illustrating RMSD, RMSF, Rg, SASA and hydrogen-bond number for the NRF2-Naringin complex. **D** Gibbs Energy Landscape diagram of MDS. **E** Representative images of CETSA showing NRF2 thermal stability after Naringin treatment. **F** CETSA curve was performed using GraphPad Prism software. N = 3/group, biological replicates. Each value was presented as the mean ± SD. ΔTm = 5.05°C

The molecular docking result revealed that the main components of TFRD-containing serum (Naringin, Eriodictyol, Esculetin, Nicotiflorin and Kaempferol 7-O-glucoside) could all effectively bind to the NRF2 protein. Each compound formed stable interactions, such as hydrogen bonds and hydrophobic interactions, at specific active sites of NRF2 (Fig. 9A). Among them, Naringin exhibited the highest binding energy with NRF2, showing the strongest binding affinity (Fig. 9B). These findings suggested that the aforementioned compounds might regulate the activity of NRF2 by directly binding to it, thereby influencing downstream antioxidant stress and other related pathways. This provided crucial clues for subsequent research on its antioxidant mechanism and potential applications.

### The serum components of TFRD could maintain the stability of NRF2

Subsequently, we selected Naringin, which had the highest binding energy, and conducted MDS with NRF2. In the MDS, after 20 ns, the RMSD values tended to stabilize, indicating that the protein reached a stable state after ligand binding. The RMSF result showed that most residues in the regions of NRF2 that directly interacted with Naringin had low RMSF values, suggesting that the protein structure was relatively stable. The Rg could reflect the compactness of the complex. After 20 ns, the Rg values decreased and stabilized, indicating that the protein structure became more compact after ligand binding. The SASA analysis revealed that after 10 ns, the SASA decreased, suggesting that the protein more tightly wrapped around the ligand, further proving their stable binding. The number of hydrogen bonds between the protein and the ligand dynamically changed during the simulation. After 10 ns, the number of hydrogen bonds stabilized, indicating that stable hydrogen bond interactions were formed between the protein and the ligand (Fig. 9C). The Gibbs Energy Landscape visually presented the free energy distribution of the complex in different conformations. Among them, the yellow/red regions were high-energy regions, representing the transition states of conformational changes. The dark blue regions represented low free energy regions (Fig. 9D), indicating that the Naringin-NRF2 complex had a relatively stable dominant conformation, which might be an important form for its biological function. These indicators revealed the interaction mode and stability characteristics of Naringin and NRF2 from different perspectives.

The CETSA verified this interaction from the perspective of cellular thermal stability. The CETSA result demonstrated that the melting temperature (Tm) of the NRF2 protein was 60.17°C in the absence of Naringin. Following treatment with Naringin, the Tm value of NRF2 significantly increased to 65.22°C, with a ΔTm value of + 5.05°C (Fig. 9E, F). This meant that after Naringin bound to NRF2, it enhanced the thermal stability of NRF2, enabling it to maintain its native conformation at higher temperatures and less prone to denaturation and aggregation. This result was highly consistent with the results of molecular docking and MDS, confirming the direct binding of Naringin and NRF2 from different levels.

## Discussion

The IMT can significantly promote the regeneration and repair of bone defects, offering hope for the repair of large segmental bone defects [24, 25]. However, despite its promising clinical outcomes, the specific osteogenic mechanisms underlying this technique, as well as how to enhance the quality and speed of osteogenesis in the induced membrane, remain unclear. Therefore, in-depth exploration of the osteogenic mechanisms of the IMT is of great significance for optimizing treatment protocols and improving the repair outcomes of bone defects.

Based on the in vivo experimental results of this study, compared with the normal bone tissue in the Sham group, iron accumulation and increased ROS metabolic products were observed in the bone tissue of the induced membrane bone graft area, indicating the occurrence of ferroptosis. This may be related to elevated local iron ion concentrations caused by trauma, as well as inflammation or ischemia–reperfusion injury, which damages the antioxidant system and leads to ROS accumulation. TFRD can dose-dependently promote the mineralization rate of bone grafts within the induced membrane, increase bone volume, and suppress the expression of ferroptosis-related factors in the bone tissue of the graft area. In vitro experiments further confirmed that TFRD exhibits significant protective effects on osteoblasts treated with the ferroptosis inducer Erastin. Specifically, TFRD reduced the accumulation of intracellular lipid peroxides (MDA, 4-HNE) and iron, while upregulating the expression of NRF2 and its downstream target genes HO-1 and GPX4. This phenomenon is highly consistent with previously reported mechanisms by which NRF2 transcriptionally activates GPX4 to eliminate lipid peroxidation [26]. Notably, osteoblasts maintained high levels of calcified nodules and osteogenesis-related factors (RUNX2 and BMP-2) after TFRD intervention, indicating that its anti-ferroptosis effect did not suppress osteogenic differentiation potential but instead provided a foundation for subsequent bone formation by maintaining cell survival. However, we found that although TFRD was similar to DMF (an NRF activator) in promoting bone graft growth in vivo and enhancing the mineralization capacity of osteoblasts in vitro, TFRD was less effective than DMF in activating NRF2-mediated anti-ferroptosis. This suggests that, in addition to its anti-ferroptosis effects, TFRD may also promote osteogenesis in the induced membrane by regulating other mechanisms. For instance, TFRD could also enhance the mineralization capacity of osteoblasts in the bone graft area of IMT through the Wnt/β-catenin and BMP/SMAD pathways [21, 27].

NRF2, as a core factor in antioxidant defense, has been extensively reported for its roles in fractures, osteoporosis, and osteoarthritis [28–30]. However, this study is the first to demonstrate the critical regulatory role of NRF2 in the microenvironment of induced membranes. The activation of the NRF2 signaling pathway is a central mechanism by which TFRD exerts its anti-ferroptosis effects. NRF2 serves as a key transcription factor in cellular responses to oxidative stress, regulating the expression of over 200 downstream genes by binding to antioxidant response elements (AREs), including GPX4 and the system Xc⁻/GSH axis, which are directly involved in ferroptosis defense [31, 32]. In this study, rats treated with TFRD exhibited enhanced NRF2 expression and its mediated anti-ferroptosis response, characterized by decreased expression of ferroptosis markers (e.g., iron, MDA, and 4-HNE) and accelerated bone defect repair. Activating NRF2 could mimic the phenotypic effects of TFRD. Meanwhile, in vitro, TFRD could reverse the promoting effect of si-NRF2 on ferroptosis in osteoblasts. These findings reveal the potential value of NRF2 as a regulatory hub for ferroptosis in the bone graft area of the induced membrane and elucidate an important mechanism by which TFRD inhibits ferroptosis in osteoblasts.

Serum pharmacochemistry is a discipline dedicated to the study of chemical substances in serum, particularly exogenous active compounds, their actions and metabolic patterns. By employing modern separation techniques and multidimensional analytical approaches, the drug components in serum can be isolated and identified [33, 34]. UHPLC-Q-Orbitrap HRMS analysis revealed that the primary compounds in serum containing TFRD were Naringin, Eriodictyol, Esculetin, Nicotiflorin and Kaempferol 7-O-glucoside, indicating the material basis for TFRD's pharmacological effects in vivo. Further investigations using molecular docking, MDS and CETSA to elucidate the mechanism by which TFRD upregulates the NRF2/ARE pathway revealed that the main component of TFRD can directly bind to NRF2 and maintain its protein stability. These findings further suggest that the mechanism by which TFRD inhibits ferroptosis in osteoblasts within the bone graft area of induced membranes is related to its direct binding to NRF2 and mediation of the NRF2/ARE pathway's signaling cascade.

Compared with previous studies, the innovations of this study are reflected in the following aspects: Firstly, most traditional Chinese medicine research has focused on the direct osteogenic effects via single signaling pathway, whereas this study reveals that TFRD achieves bone regeneration by regulating NRF2-mediated antioxidant and anti-ferroptosis actions. For example, previous studies have reported that TFRD can promote osteogenic differentiation of osteoblasts in the bone graft area by activating the Wnt/β-catenin or BMP/SMAD pathways, but they did not explain its protective effects under oxidative stress conditions [21, 27]; in contrast, this study supplements its new role in ferroptosis regulation and enhances the theory regarding TFRD-mediated multi-pathway regulation for induced membrane bone regeneration. Finally, this study provides a natural drug solution for optimizing the IMT. Currently, the limitations of this technique lie in the insufficient donor supply for autologous bone grafting in the second stage and the long osteogenic cycle. As a natural product with low immunogenicity, multi-target synergy, and low cost, TFRD, when administered orally or delivered locally (e.g., loaded onto bone cement scaffolds), may emerge as a novel strategy to assist or even replace autologous bone grafting. Additionally, the inhibitory effect of TFRD on ferroptosis may extend to other ferroptosis-related diseases (such as ischemic osteonecrosis, post-radiotherapy bone injury, osteoporosis, etc.), demonstrating broad application prospects.

The limitations of this study lie in the fact that the bone metabolism rate of the rat model used in the experiments differs from that of humans, necessitating validation of result reproducibility in large animals (such as pigs or sheep). Although the experiments confirmed the necessity of NRF2 in ferroptosis within the bone graft area, the possibility that TFRD may exert synergistic effects through other pathways (such as FSP1/CoQ10/NAD(P)H, DHODH/CoQH2, AMPK, etc.) has not been ruled out. Additionally, the long-term toxicity assessment of the drug is insufficient, and it is essential to monitor the effects of high-dose or long-term TFRD use on liver and kidney function. Finally, future research should focus on developing targeted delivery systems for TFRD (such as nanoparticles or hydrogels) to enhance its local concentration and retention time at the bone defect site. Concurrently, single-cell sequencing technology should be employed to analyze the differential responses of various cell types within the induced membrane (such as osteoblasts, vascular endothelial cells, and macrophages) to TFRD.

## Conclusion

The anti-ferroptosis effect mediated by NRF2 in osteoblasts represents one of the mechanisms underlying the repair of large bone defects using the IMT. The primary active components of TFRD maintain NRF2 stability by targeting and binding to it, thereby activating the NRF2/ARE pathway and inhibiting ferroptosis in osteoblasts. This, in turn, improves the quality of bone graft mineralization within the induced membrane and promotes the repair of large bone defects. This discovery not only enhances our understanding of the bone regeneration microenvironment in the IMT, but also provides experimental evidence for the development of natural product-based adjuvant strategies for the IMT.

## Supplementary Information

Supplementary Material 1

Supplementary Material 2

## Data Availability

Data will be made available on request.

## Abbreviations

IMT: Induced membrane technique
TFRD: Total flavonoids of *Rhizoma* *drynariae*
PMMA: Polymethylmethacrylate
RUNX2: Runt-related transcription factor 2
ROS: Reactive oxygen species
NRF2: Nuclear factor erythroid 2-related factor 2
ARE: Antioxidant response element
GPX4: Glutathione peroxidase 4
HO-1: Heme oxygenase 1
NQO1: NAD(P)H quinone oxidoreductase 1
GSH: Glutathione
system Xc: Cystine/glutamate antiporter
SLC7A11: Solute carrier family 7 member 11
SD: Sprague-Dawley
Micro-CT: Micro computed tomography
BV/TV: Bone volume fraction
HE: Hematoxylin-eosin
SO-FG: Safranin O-fast green
VEGF: Vascular endothelial growth factor
BMP2: Bone morphogenetic protein 2
DMEM: Dulbecco's modified eagle medium
FBS: Fetal bovine serum
OD: Optical density
ALP: Alkaline Phosphatase
ARS: Alizarin Red S
MDA: Malondialdehyde
4-HNE: 4-Hydroxy-2-nonenal
ELISA: Enzyme-linked immunosorbent assay
TEM: Transmission electron microscopy
qRT-PCR: Quantitative real time-polymerase chain reaction
LC-MS: Liquid chromatography-mass spectrometry
MDS: Molecular dynamics simulation
RMSD: Root mean square deviation
RMSF: Root mean square fluctuation
Rg: Radius of gyration
SASA: Solvent-accessible surface area
CETSA: Cellular thermal shift assay
Tm: Melting temperature
