# Supplementary figures and images for: Total flavonoids of Rhizoma drynariae targets NRF2-mediated anti-ferroptosis in osteoblasts to promote induced membrane osteogenesis
Source: Chin Med. 2026 Mar 13;21:83. doi: 10.1186/s13020-026-01347-7 (PMC12983768; doi:10.1186/s13020-026-01347-7)

1

2

3

4

5

6

7


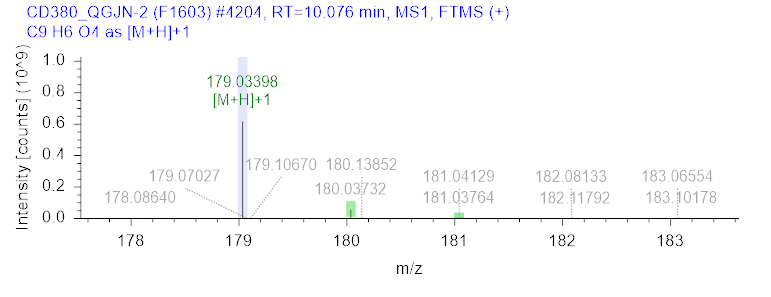


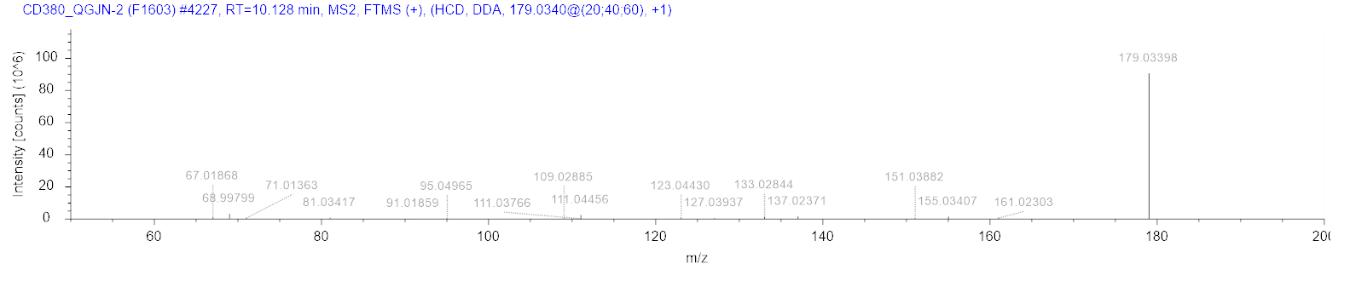


8

9

10

11

12

13

14


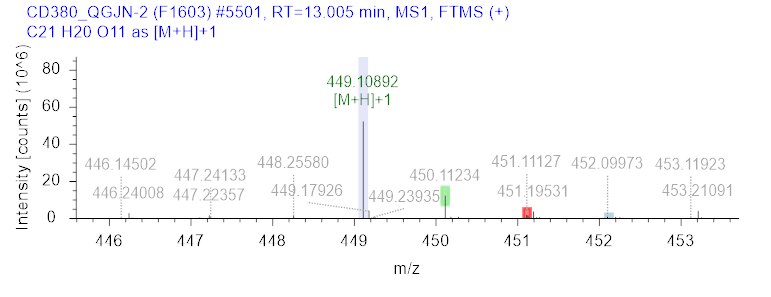


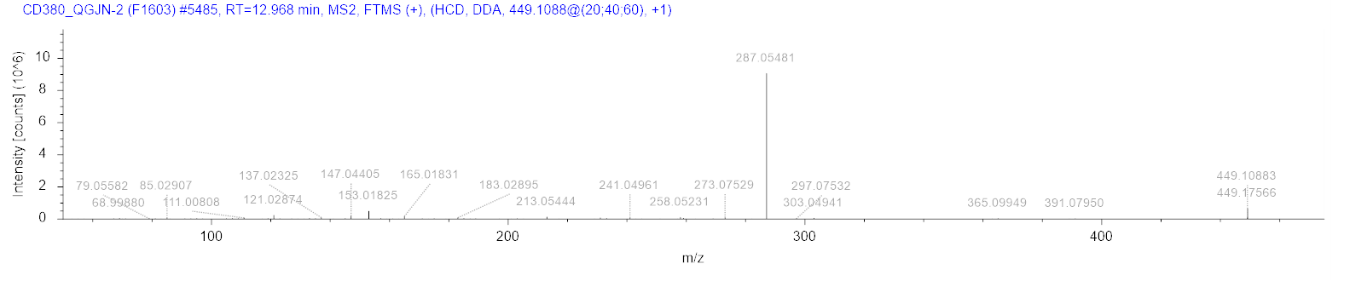


15


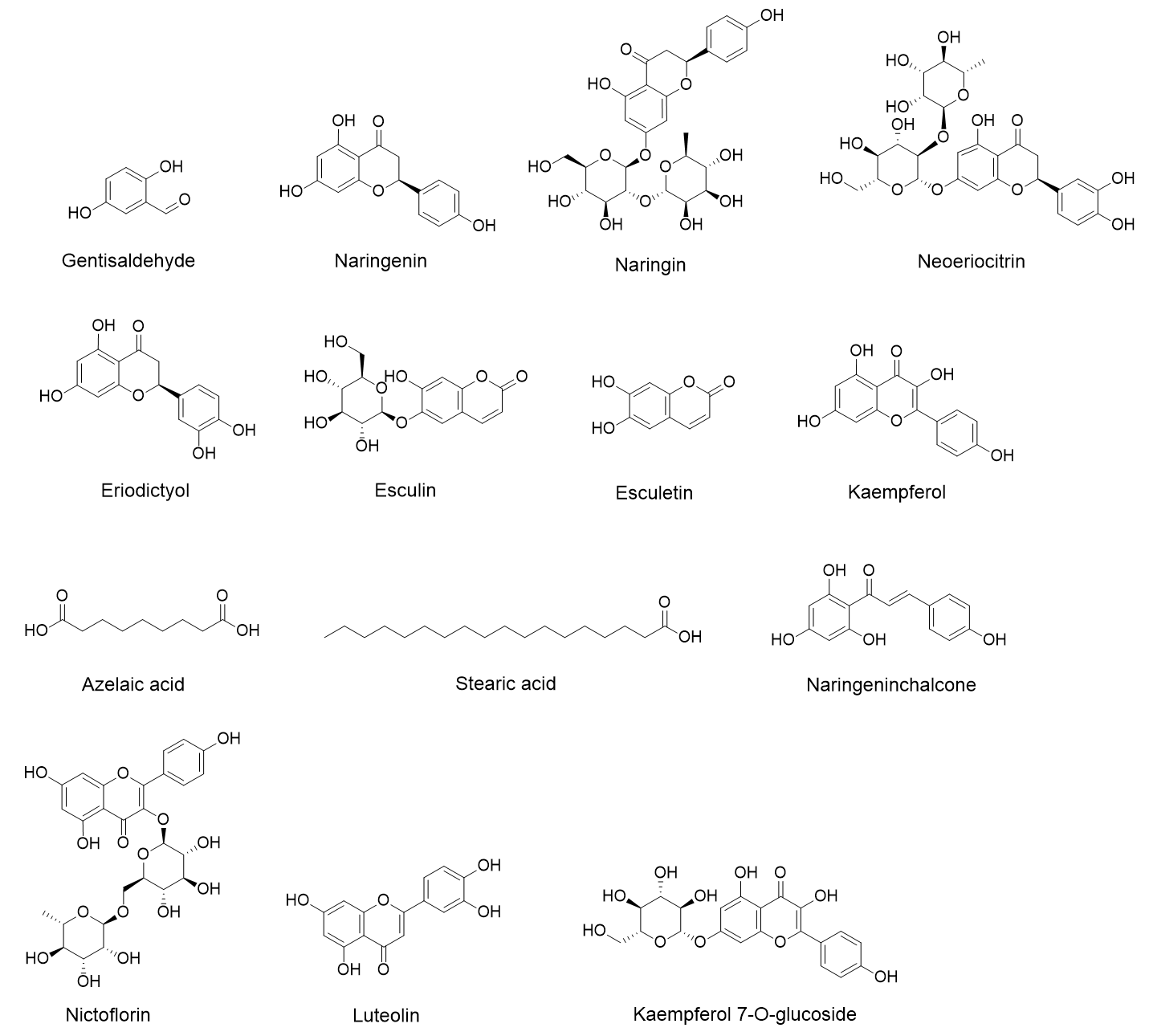

Supplement: Supplementary file 1 — Supplementary Material 1 [file 13020_2026_1347_MOESM1_ESM.docx]
