# Supplementary material for: Total flavonoids of Rhizoma drynariae targets NRF2-mediated anti-ferroptosis in osteoblasts to promote induced membrane osteogenesis
Source: Chin Med. 2026 Mar 13;21:83. doi: 10.1186/s13020-026-01347-7 (PMC12983768; doi:10.1186/s13020-026-01347-7)
Supplement: Supplementary file 2 — Supplementary Material 2 [file 13020_2026_1347_MOESM2_ESM.docx]

| Project | Parameter |
| --- | --- |
| Ion Source | Electrospray Ionization (ESI) |
| Scan Mode | Positive and Negative Ion Switching Scan |
| Detection Mode | Full mass/dd-MS_2_ |
| Resolution | 70000（full mass）；17500（dd-MS_2_） |
| Scan Range | 100.0～1500.0 m/z |
| Spary Voltage | 3.2 kV（Positive, Negative） |
| Capillary Temperature | 300 ℃ |
| Collision Gas | High-Purity Argon (Purity ≥ 99.999%) |
| Collision Energy(N)CE | 30，40，60 |
| Sheath Gas | Nitrogen (Purity ≥ 99.999%), 40 Arb |
| Auxiliary Gas | Nitrogen (Purity ≥ 99.999%), 15 Arb, 350 ℃ |

**Table 1**

| Project | Parameter |
| --- | --- |
| Chromatographic Column | AQ-C18，150×2.1mm，1.8 µm，Welch |
| Flow Rat | 0.30 mL/min |
| Aqueous Phase | 0.1% Formic Acid in Wate |
| Organic Phase | Methanol |
| Column Oven Temperature | 35 ℃ |
| Autosampler Temperature | 10.0℃ |
| Injection Volume (Autosampler) | 5.00 µL |

**Table 2**

| Peak No. | tR/min | Chemical formula | Theoretical mass | Observed mass | Error (ppm) | MS/MS fragments | Identification |
| --- | --- | --- | --- | --- | --- | --- | --- |
| 1 | 8.4 | C_7_H_6_O_3_ | 137.0244 | 137.0232 | -8.8 | 91.0176、117.8772、108.0203、137.0233 | Gentisaldehyde |
| 2 | 14.5 | C_15_H_12_O_5_ | 271.0612 | 271.0615 | -0.2 | 107.0125、119.0490、151.0026、271.0615 | Naringenin |
| 3 | 12.5 | C_27_H_32_O_14_ | 579.1719 | 579.1718 | -0.2 | 151.0026、271.0615、459.1155、579.1724 | Naringin |
| 4 | 11.3 | C_27_H_32_O_15_ | 597.1814 | 597.1828 | 2.3 | 289.0707、459.1155、597.2417 | Neoeriocitrin |
| 5 | 13.6 | C_15_H_12_O_6_ | 287.0561 | 287.0565 | 1.4 | 135.0440、151.0026、243.0666、287.0554 | Eriodictyol |
| 6 | 10.1 | C_15_H_16_O_9_ | 341.0867 | 341.0869 | 0.6 | 133.0285、179.0339、341.1493 | Esculin |
| 7 | 10.1 | C_9_H_6_O_4_ | 179.0339 | 179.0340 | 0.6 | 123.0443、133.0284、151.0388、179.0340 | Esculetin |
| 8 | 14.3 | C_15_H_10_O_6_ | 287.0550 | 287.0556 | 2.1 | 165.0184、213.0549、241.0502、287.0552 | Kaempferol |
| 9 | 13.4 | C_9_H_16_O_4_ | 187.0976 | 187.0969 | -3.4 | 97.0645、125.0960、126.0993、187.0969 | Azelaic acid |
| 10 | 24.0 | C_18_H_36_O_2_ | 283.2643 | 283.2648 | 1.8 | 239.2013、283.2646 | Stearic acid |
| 11 | 12.5 | C_15_H_12_O_5_ | 273.0758 | 273.0757 | -0.4 | 147.0440、153.0182、273.0757 | Naringeninchalcone |
| 12 | 11.5 | C_27_H_30_O_15_ | 595.1658 | 595.1663 | 0.8 | 287.0549、449.1078 | Nicotiflorin |
| 13 | 16.2 | C_15_H_10_O_6_ | 285.0405 | 285.0410 | 1.8 | 107.0126、133.0282、151.0025、285.0404 | Luteolin |
| 14 | 13.0 | C_21_H_20_O_11_ | 449.1078 | 449.1089 | 2.4 | 、287.0548、449.1088 | Kaempferol 7-O-glucoside |

**Table 3**
